# A Convenient, Pd-Free Approach to the Synthesis of Risdiplam

**DOI:** 10.3390/molecules30163375

**Published:** 2025-08-14

**Authors:** Georgiy Korenev, Alexey A. Gutenev, Fyodor V. Antipin, Vladimir V. Chernyshov, Julia A. Shulgina, Maria P. Korobkina, Maxim B. Nawrozkij, Roman A. Ivanov

**Affiliations:** Medicinal Biotechnology Department, Sirius University of Science and Technology, Olimpiyskiy Ave. 1, 354340 Sirius, Krasnodar Region, Russiavladimir.chernyshov2012@yandex.ru (V.V.C.); navrotskij.mb@talantiuspeh.ru (M.B.N.); ivanov.ra@talantiuspeh.ru (R.A.I.)

**Keywords:** risdiplam, spinal muscular atrophy (SMA), straightforward synthesis, heterocyclization

## Abstract

Several approaches to the synthesis of risdiplam, a pharmacologically relevant pyridopyrimidinone derivative, have been recently reported. However, most of these routes rely exclusively on palladium-catalyzed, cross-coupling reactions and involve low-yielding intermediates, which limit their scalability and complicate impurity control. In this work, we present a five-step, straightforward route to risdiplam, utilizing ethyl 2,8-dimethylimidazo[1,2-*b*]pyridazine-3-carboxylate—an accessible and cost-effective building block previously developed by our research group—as a starting material. The key step involves construction of the 4*H*-pyrido[1,2-*a*]pyrimidin-4-one scaffold via a copper(I)-catalyzed heterocyclization reaction. This represents a novel and convenient protocol for the synthesis of 2-(2,8-dimethylimidazo[1,2-*b*]pyridazin-6-yl)-7-fluoro-4*H*-pyrido[1,2-*a*]pyrimidin-4-one, which serves as a crucial intermediate in the final stages of risdiplam synthesis. The overall process allows us to obtain the target compound with a 20% total yield (from abovementioned starting material) and high purity (99.86%, by HPLC-UV), with a maximum level of unidentified impurities not exceeding 0.046%. The developed approach eliminates the use of palladium catalysis and chromatographic purification, offering a practical and scalable alternative for risdiplam production.

## 1. Introduction

Spinal muscular atrophy (SMA) is a severe progressive neuromuscular disorder that can lead to premature death in both pediatric and adult patients [1,2,3]. Over 95% of cases are attributed to an autosomal recessive condition caused by homozygous deletions or mutations in the *survival motor neuron 1* (*SMN1*) gene located on chromosome 5q13 [4,5]. While humans possess a paralogous gene, *SMN2*, which also encodes the SMN protein, a critical difference in exon 7 results in its exclusion during mRNA splicing. Consequently, *SMN2* produces only limited amounts of functional SMN protein, insufficient to compensate for the loss of *SMN1* expression in patients with SMA.

To date, several disease-modifying therapies have been approved by the U.S. Food and Drug Administration (FDA) for the treatment of SMA. These include nusinersen, an intrathecally administered antisense oligonucleotide that modulates *SMN2* splicing, and onasemnogene abeparvovec, a one-time intravenous gene therapy based on an adeno-associated viral (AAV9) vector delivering a functional copy of *SMN1* [6,7,8].

The only small-molecule therapeutic currently approved for SMA is risdiplam, an orally administered compound that promotes inclusion of exon 7 in *SMN2* transcripts, thereby enhancing production of full-length SMN protein [9,10,11]. Clinical trials have demonstrated that risdiplam provides sustained therapeutic benefit across a broad and clinically heterogeneous patient population with later-onset SMA. After 24 months of treatment, 32% of patients showed improvement in motor function, while 58% maintained stable scores according to the Motor Function Measure 32 (MFM32) scale [12,13,14]. These findings highlight risdiplam’s clinical value in the evolving therapeutic landscape of SMA [15], while also underscoring the need for alternative synthetic strategies and broader access to ensure long-term availability and health systems resilience.

**Figure 1:**
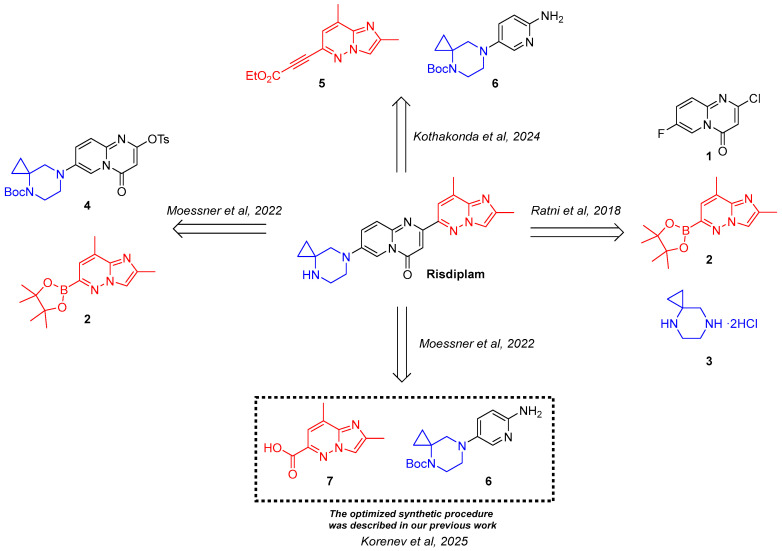
Four distinct retrosynthetic strategies for the risdiplam synthesis [16,17,18,19]; the imidazo[1,2-*b*]pyridazine and 4,7-diazaspiro[2.5]octane fragments present in the risdiplam molecule are highlighted in red and blue, respectively, with the corresponding colors also used to indicate the key building blocks employed for incorporating the abovementioned fragments into the final structure of the target molecule.

Up to date, several synthetic approaches to risdiplam have been reported, differing in their underlying retrosynthetic logic and the sets of key intermediates involved (Figure 1). Notably, each strategy employs a distinct method for the construction of the central 4*H*-pyrido[1,2-*a*]pyrimidin-4-one scaffold, which serves as the defining structural motif of the molecule.

The pioneer synthetic scheme, proposed by Ratni and co-authors [18], employs a Conrad–Limpach-type condensation, followed by a deoxychlorination step to construct the 2,7-disubstituted 4*H*-pyrido[1,2-*a*]pyrimidin-4-one core **1**. The resulting intermediate is then decorated via the sequential interactions with building blocks **2** and **3** to afford the target molecule. Subsequently, an optimized commercial synthesis of risdiplam has been described by Moessner and co-workers [16], which implements a similar conceptual framework. However, in this route, the final cross-coupling reactions involve a pre-functionalized pyridopyrimidinone **4** and a coupling-ready imidazopyridazine motif **2**, streamlining the late-stage assembly of the molecule.

The other two approaches rely on the assembly of the central scaffold from fragments comprising a substituted aminopyridine **6** and a C-3-functionalized imidazo[1,2-*b*]pyridazine moiety **5** and **7**. For example, researchers from Biophore India Pharmaceuticals Pvt Ltd. (Telangana, India) proposed the use of a propiolate-containing fragment **5** as a condensation agent in the key step [17]. Meanwhile, Moessner and colleagues [16], at the initial stages of optimization process, developed a strategy based on the 2,8-dimethylimidazo[1,2-*b*]pyridazine-6-carboxylic acid **7** and its subsequent derivative with Meldrum’s acid, but due to the lack of stable intermediates amenable to crystallization, it was not selected for the further development of a commercial route for the production of risdiplam.

**Figure 2:**
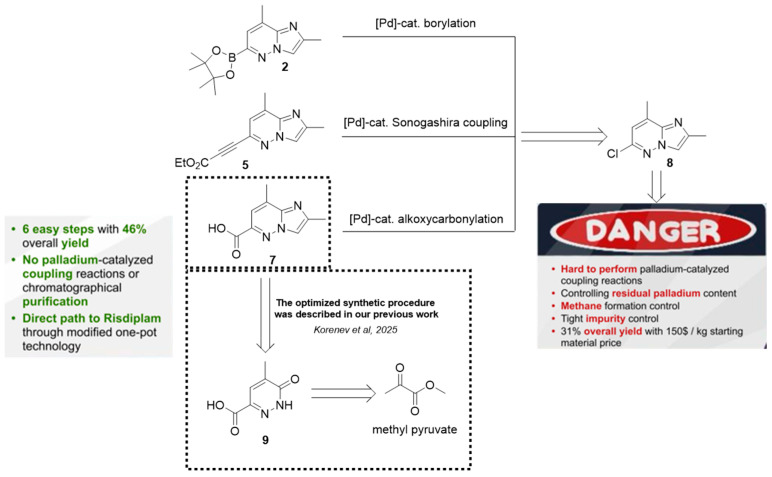
Challenging approaches to intermediates **2**, **5**, and **7** synthesis and the earlier presented alternative, which eliminates all the characteristic shortcomings [19].

Notably, the synthesis of compounds **2**, **5,** and **7** as well as original strategies that utilize them as the key intermediates, rely exclusively on cascades of palladium complex-catalyzed cross-coupling reactions, albeit with varying degrees of complexity. Moreover, the synthesis of a precursor for all of the abovementioned key intermediates—6-chloro-2,8-dimethylimidazo[1,2-*b*]pyridazine **8** (Figure 2)—also involves a hard-to-perform Pd complex-catalyzed modified Negishi coupling as a key step, which presents several challenges, including the need for the strict control over methane formation and by-product removal. Moreover, stringent specifications for impurity levels, residual palladium content, and the modest overall yield (31%) [16] make the preparation of all these intermediates a demanding task (Figure 2).

All of these limitations have been fully addressed by our research group, which developed an original, practical, and cost-efficient synthetic route to intermediate **7** (Figure 2). This method proceeds from readily available starting materials and entirely avoids the use of palladium complex-catalyzed cross-coupling reactions or chromatographic purifications, thereby simplifying the process and improving overall efficiency [19]. Moreover, our group developed a modified route to risdiplam, based on previously described strategies, utilizing a 2,8-dimethylimidazo[1,2-*b*]pyridazine-6-carboxylic acid **7** as a key intermediate (Figure 1) [19]. The subsequent heterocyclization proceeded via a modified one-pot Meldrum’s acid-based strategy, with a stable and crystalline check-point, enabling efficient construction of the target heterocyclic core.

**Figure 3:**
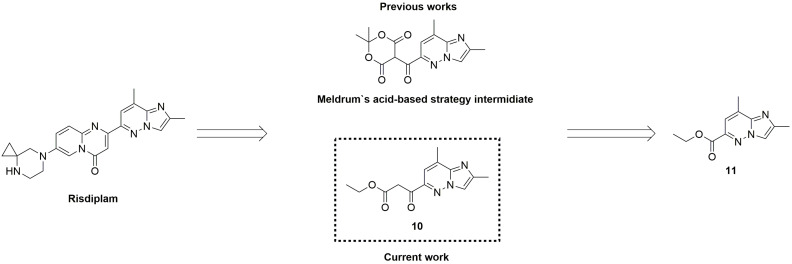
The proposed retrosynthetic pathway to risdiplam employing ethyl 3-(2,8-dimethylimidazo[1,2-b]pyridazin-6-yl)-3-oxopropanoate **10** as the key intermediate.

However, even the modified late-stages protocol previously reported, presents several limitations. Specifically, the process still lacks sufficient number of check-points, exhibits low atom economy, generates a significant amount of waste, including substantial quantities of inorganic by-products and involves the extensive use of chlorinated organic solvents and reagents—recognized as persistent environmental pollutants. These limitations prompted us to develop an alternative synthetic strategy (Figure 3) for risdiplam, designed to overcome the aforementioned issues.

Notably, Meldrum’s acid-based strategies are not the only ones in which imidazopyridazine carboxylic acid derivatives can serve as a starting material (Figure 3). In order to remove 2,2-dimethyl-5-[(2,8-dimethylimidazo[1,2-b]pyridazin-6-yl)(hydroxy)methylidene]-1,3-dioxane-4,6-dione and its moderately stable deoxychlorination product in the synthetic scheme, a possibility of direct cyclocondensation of ethyl 3-(2,8-dimethylimidazo[1,2-*b*]pyridazin-6-yl)-3-oxopropanoate **10** with the proper 2-aminopyridine was taken into consideration for the construction of the required 4*H*-pyrido[1,2-*a*]pyrimidin-4-one derivative. Thus, the possibility of a straightforward synthesis of compound **10**—for example, via direct Claisen condensation from the corresponding ester **11**—together with optimized and scalable procedures for the preparation of the latter, supports the consideration of the presented β-ketoester **10** as a key intermediate for the design and implementation of a new synthetic route to risdiplam.

## 2. Results and Discussion

Previously, we proposed an effective protocol for the preparation of ethyl 2,8-dimethylimidazo[1,2-*b*]pyridazine-6-carboxylate **11** as an alternative building block for risdiplam synthesis [19]. The present study is devoted to the use of the ester **11** as a starting material in a completely new approach to the synthesis of risdiplam.

**Figure 4:**
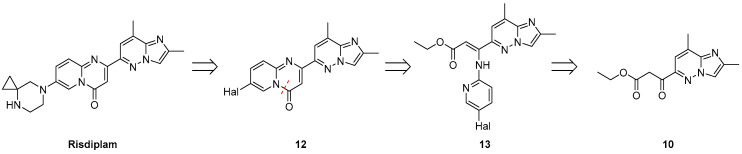
Retrosynthetic analysis of the risdiplam, based on C-4/N-5 bond disconnection (red dotted line) within the structure of 4*H*-pyrido[1,2-*a*]pyrimidin-4-one core **12**.

The first step has been devoted to the construction of the central 7-halogeno-2-(2,8-dimethylimidazo[1,2-*b*]pyridazin-6-yl)-4*H*-pyrido[1,2-*a*]pyrimidin-4-one core **12** (Figure 4). In contrast to many literature-reported methods, which mostly relies on the Conrad–Limpach-type reactions, followed by subsequent Pd complex-catalyzed cross-couplings, the synthesis of the target intermediate **12** can also be achieved via a Gould–Jacobs-type condensation. Retrosynthetic disconnection of the C-4/N-5 bond reveals the corresponding enamino-ester **13** as a viable synthetic intermediate. However, the preparation of this intermediate, while conceptually simple, presents, in fact, non-trivial challenges due to multiple possible routes and selectivity issues.

The most evident and common approach to **13** involves direct condensation between the proper 2-aminopyridine derivative and β-ketoester **10**. The latter has been prepared according to a variation in the known protocol [20] via condensation of the ester **11** with EtOAc in the presence of LDA at reduced temperatures, resulting in a product **10** with a high purity and yield. Since no literature precedent was available for the next condensation reaction using novel substrate **10** and the synthesis of the compound **13** has never been previously described, we tried to carry out this transformation in the presence of TsOH, Bi(OTf)_3_ or InBr_3_, because it is well-known (for example [21,22,23]) that similar condensations may be effectively catalyzed by protic or aprotic Lewis acids. However, neither approach afforded the desired intermediate. Utilization of TsOH in a set of high-boiling solvents (toluene, anisole, nitrobenzene) consistently led to the formation of 1-(2,8-dimethylimidazo[1,2-*b*]pyridazin-6-yl)ethan-1-one **14** (see Experimental Section and ESI p. 5) in approximately 70% isolated yield (from the synthesis with TsOH in toluene). It is worth noting that no conversion of the starting β-ketoester **10** was observed below 100 °C. On the other hand, the use of aprotic Lewis acids such as bismuth triflate or indium bromide presumably resulted in the formation of N-(5-bromopyridin-2-yl)-3-(2,8-dimethylimidazo[1,2-*b*]pyridazin-6-yl)-3-oxopropanamide as a major product (the structure was suggested by HPLC-UV-MS analysis, considering the observed molecular ion mass and the characteristic isotopic pattern). Unsuccessful results on the tested substrates may be due to the sterical hindrance of the keto-group in the substrate **10**, accompanied by the Coulomb repulsion between the nitrogen atom in the side chain of 2-aminopyridine derivative and a pyridazine nitrogen lone pair of electrons.

The alternative strategy involved combining the 2-amino-5-bromopyridine and a halogen-bearing component. We attempted to synthesize a substituted ethyl 3-chloroacrylate from the corresponding β-ketoester **10**, aiming to later displace the halogen atom and induce heterocyclization. However, oxalyl chloride—recognized here as a mild deoxychlorinating agent—proved completely ineffective for this purpose, while phosphorus oxychloride failed to initiate the reaction at room temperature and caused decomposition of the starting material at 50–60 °C.

**Figure 5:**
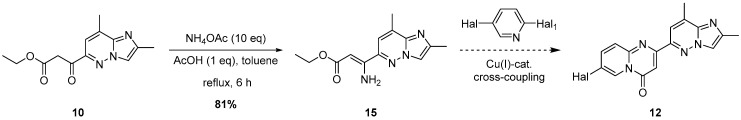
The novel robust pathway to the 4*H*-pyrido[1,2-*a*]pyrimidin-4-one core **12**.

To overcome all these difficulties, we decided to perform a castling between the nucleophilic and an electrophilic reagent. So, we substituted 2-aminopyridine derivative for 2-halogenpyridine and, simultaneously, exchanged β-ketoester **10** for the corresponding 3-aminoacrylate **15** (Figure 5). Compound **15**, in turn, was obtained in a high yield by treatment of the β-ketoester **10** with NH_4_OAc and AcOH in refluxing toluene.

The selection of an appropriate dihalogenated pyridine was guided by the following considerations: the halogen at the 2-position (Hal_1_) of the pyridine ring was expected to readily undergo copper complex-catalyzed substitution, while the halogen at the 5-position was anticipated to remain intact under the same conditions. The latter should, however, retain the potential to be subsequently substituted by the spirocyclic amine moiety in a non-catalytic manner. As the solvent and temperature parameters had already been thoroughly optimized on simpler substrates for this type of copper-catalyzed heterocyclization in a previously published study by Mo et al. [24], we decided to keep these conditions constant. Moreover, although potassium bicarbonate was selected as the base of choice in the abovementioned study, we chose to replace it with potassium carbonate, which is, in fact, the actual base in the reaction, being formed from the corresponding acidic salt upon heating under the reaction conditions. Consequently, the selection of the heterocyclization conditions was focused exclusively on identifying the most suitable substrate and the most efficient ligand (Table 1).

**Figure 6:**
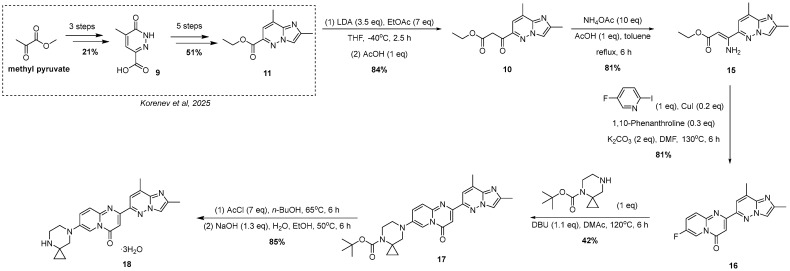
The novel scheme of risdiplam trihydrate synthesis [19].

As a result, 2-iodo-5-fluoro-pyridine was selected as a substrate and 1,10-phenanthroline was a ligand of choice for this synthesis. Despite the fact that, during the original investigation [24], phosphine-based ligands, in general, and MePhos, in particular, gave the best results in terms of yield of the title compounds, our results are in good agreement with the results on catalytic amination of 2-halogenpyridines by Beletskaya and co-authors [25,26]. After successful assembly of the target intermediate **16** (Figure 6), the remaining steps toward risdiplam became straightforward.

Historically, throughout the development of the risdiplam synthetic route, the substitution of fluorine atom in **16** by the spirocyclic amine fragment has consistently posed significant challenges, typically proceeding with very low yields. Several of these issues have been addressed. For example, regioselectivity was improved by employing *tert*-butyl 4,7-diazaspiro[2.5]octane-4-carboxylate instead of the unprotected analog, and replacing DMSO (which exhibits oxidative properties at elevated temperatures) with *N,N*-dimethylacetamide as the solvent. Nevertheless, the rate of the substitution reaction using *tert*-butyl 4,7-diazaspiro[2.5]octane-4-carboxylate remains among the lowest observed in a series of structurally related amines, probably due to 1,3-diaxial interactions [16]. As a result, prolonged heating at elevated temperatures leads to partial resinification of the reaction mixture, causing a substantial decrease in the overall yield. However, non-catalytic substitution of the fluorine atom with the appropriately *tert*-butyl 4,7-diazaspiro[2.5]octane-4-carboxylate, albeit proceeding in moderate yield, followed by subsequent deprotection, furnished the corresponding risdiplam dihydrochloride. The final conversion to the free base was carried out using previously reported methods [16]. The resulting risdiplam was obtained with a purity of 99.86% (by HPLC-UV), with a maximum content of a single, unidentified impurity not exceeding 0.046% (see ESI, p. 20).

Finally, a convenient and straightforward five-step synthetic route to risdiplam was developed, starting from ethyl 2,8-dimethylimidazo[1,2-*b*]pyridazine-3-carboxylate **11**—an accessible and readily prepared intermediate. The initial part of this study describes a completely new three-step protocol for the synthesis of 2-(2,8-dimethylimidazo[1,2-*b*]pyridazin-6-yl)-7-fluoro-4*H*-pyrido[1,2-*a*]pyrimidin-4-one **16**, which enables the preparation of the target compound with a 20% total yield (from **11**) and high purity (99.86%, by HPLC-UV). A key feature of this protocol is that it represents the first reported method for the synthesis of compound **16** (and potentially its analogs) that entirely avoids Pd complex-catalyzed transformations (Figure 6). Furthermore, the proposed approach ensures process transparency through intermediate isolation and enables tight impurity specifications, rendering it amenable to scaling up. In addition, improved atom economy, the reduction in inorganic waste, and the establishment of robust control points address major limitations of previously reported methods, positioning this approach as a more environmentally sustainable alternative.

## 3. Materials and Methods

### 3.1. General

All solvents and reagents were obtained from commercial sources and used without further purification unless otherwise stated. ^1^H and ^13^C NMR spectra were recorded on a Bruker Avance Neo 400 MHz spectrometer (Mannheim, Germany, 400.1, 100.6 MHz, respectively) in CDCl_3_, DMSO-*d*_6_ solutions. Chemical shifts δ are reported in parts per million (ppm); multiplicity: *s*, singlet; *d*, doublet; *t*, triplet; *q*, quartet; *dd*, double of doublets; *m*, multiplet; *br*, broad; the coupling constants *J* are reported in Hz. The structure of the products was determined by analyzing ^1^H and ^13^C NMR spectra; assignments on a routine basis by a combination of 1D and 2D experiments (HSQC, HMBC). HPLS-UV-MS analyses were performed on a «Vanquish Flex» chromatograph (Thermo Scientific, Waltham, MA, USA) with Diode Array Detector FG (DAD FG, Thermo Scientific, USA) combined with an ISQ EM Single Quadrupole Mass Spectrometer (Thermo Scientific, USA). A 6-minute gradient separation on an Agilent Poroshell 120 EC-C18 (Santa Clara, CA, USA, 100 mm × 2.0 mm, particle size 1.9 μm) column was run under the following conditions: Solvent A = water with 0.1% formic acid, solvent B = acetonitrile with 0.1% formic acid, from 0 to 2.5 min; gradient elution from A:B = 9:1 to A:B = 1:9, from 2.5 min to 3.5 min; elution in A:B = 1:9, from 3.5 min to 6 min; equilibration of the chromatographic column in A:B = 19:1 at a flow rate 0.5 mL/min. Column temperature was 40 °C, and injection volume of the sample was 1 µL. An electrospray ionization source was used to ionize the samples. Ions of positive and negative polarities were detected in the full ion current recording mode; the range of recorded masses was 10–700 *m*/*z*. The absorption spectra were recorded on a diode array detector at 2 wavelengths: 220 nm and 254 nm. High-resolution mass spectrometry (HRMS) analyses were performed using a Bruker maXis II 4G ETD mass spectrometer and an UltiMate 3000 chromatograph (Thermo Scientific, Waltham, MA, USA) equipped with Acclaim RSLC 120 C18 2.2 μm 2.1 × 100 mm column. Spectrum registration mode was electrospray ionization (ESI), with a full scan between *m*/*z* 100 and 1500, tandem MS (MS/MS) with selection of three most intense ions, collision-induced dissociation (CID) at 10–40 eV, and nitrogen as a collision gas. Melting points were determined on Melting Point Apparatus SMP50 (Norrscope, Bicknacre, UK) in a 1 °C/minute regime. The target substances were lyophilized using a LABCONCO FreeZone 2.5 L freeze dryer (Labconco, Kansas City, MO, USA, samples were preliminarily frozen in a freezer at −80 °C for 5 h, sublimation was carried out for 12 h, with residual pressure of 0.003 mbar). Thin-layer chromatography (TLC) was carried out on Merck silica gel 60 F254 precoated plates (Darmstadt, Germany); compounds on TLC were visualized by illumination under UV light (254 nm) or by ninhydrin or phosphomolybdic acid staining.

### 3.2. Analysis of the Risdiplam Purity

HPLS-UV analyses were performed on a «Prominence-I LC-2030C Plus» chromatograph (Shimadzu, Kyoto, Japan). A 40 min gradient separation on an Agilent Poroshell 120 EC-C18 (100 mm × 4.6 mm, particle size 2.7 μm, with a pore size of 120 Ǻ) column was run under the following conditions: solvent A = water with 0.1% orthophosphoric acid, adjusted to pH 3.5 with triethylamine, and solvent B = acetonitrile (gradient for HPLC) from 0 to 7 min; gradient elution from A:B = 7.3:2.7 to A:B = 7.0:3.0 from 7 to 17 min; elution in A:B = 6.9:3.1 from 17 to 20 min; elution in A:B = 6.5:3.5 from 20 min to 30 min; elution in A:B = 0.5:9.5 from 30 to 31 min; elution in A:B = 0.5:9.5 from 31 to 35 min; elution in A:B = 7.3:2.7 from 35 to 40 min; equilibration of the chromatographic column in A:B = 7.3:2.7 at a flow rate 0.5 mL/min. Column temperature was 30 °C, autosampler temperature was 8 °C, and injection volume of the sample was 5 µL. The absorption spectra were recorded on a UV detector at a wavelength of 258 nm.

### 3.3. Synthesis of Ethyl 2,8-Dimethylimidazo[1,2-b]pyridazine-6-carboxylate **11**

An ethyl 2,8-dimethylimidazo[1,2-*b*]pyridazine-6-carboxylate **11** was synthesized from methyl pyruvate according to the protocols described earlier [19].

### 3.4. Synthesis of Ethyl 3-(2,8-dimethylimidazo[1,2-b]pyridazin-6-yl)-3-oxopropanoate **10**

A well-stirred solution of ethyl 2,8-dimethylimidazo[1,2-*b*]pyridazine-6-carboxylate **11** (47.4 g, 216 mmol, 1 eq) in a mixture of absolute THF (200 mL) and absolute EtOAc (150 mL, 1.515 mol, 7 eq) under an argon-blanket was cooled to −40 °C and treated with a commercially available 2M solution of LDA in THF-PhEt-C_7_H_16_ mixture (380 mL, 756 mmol, 3.5 eq), gradually warmed to 25 °C, and kept for 1.5 h at this temperature. After reaction finished (TLC—control on starting material, eluent—EtOAc 100%, R_f_ 0.55), the reaction mixture was treated with glacial AcOH until the formation of a clear solution was observed and quenched with deionized water (200 mL). The organic layer was separated and the aqueous layer was extracted with EtOAc (3 × 200 mL). The combined organic layers were washed with brine (2 × 150 mL), dried over anhydrous Na_2_SO_4_, and filtered and stripped down from the solvent in vacuo. The residue was dissolved in a minimal volume of EtOAc and the target ethyl-3-(2,8-dimethylimidazo[1,2-*b*]pyridazine-6-yl)-3-oxopropionate 10 was precipitated with *n*-hexane as a light beige powder, which was filtered off and air-dried to constant weight. Yield—42.3 g (84%).

^1^H NMR (400 MHz, DMSO-*d*_6_, *δ*): 8.16 (^1^H, *d*, *J* = 1.0 Hz, 7-CH), 7.52 (^1^H, *d*, *J* = 1.2 Hz, 3-CH), 4.19 (^2^H, *s*, EtO_2_CCH_2_CO-), 4.13 (^2^H, *q*, *J* = 7.1 Hz, CH_2_CH_3_), 2.60 (^3^H, *d*, *J* = 1.1 Hz, 2-CH_3_), 2.43 (^3^H, *d*, *J* = 0.9 Hz, 8-CH_3_), 1.17 (^3^H, *t*, *J* = 7.1 Hz, CH_2_CH_3_). ^13^C NMR (101 MHz, DMSO-*d*_6_, *δ*): 192.1 (6-COOCH_2_CO_2_Et), 167.9 (6-COOCH_2_CO_2_Et), 146.6 (C-6), 145.7 (C-9), 139.9 (C-2), 136.6 (C-8), 115.4 (C-3), 113.4 (C-7), 61.2 (COOCH_2_CH_3_), 44.5 (6-COOCH_2_CO_2_Et), 16.6 (2-CH_3_), 15.1 (8-CH_3_), 14.4 (COOCH_2_CH_3_). HRMS (ESI+): found *m*/*z* 262.1210 [M + H]^+^; calculated for C_13_H_16_N_3_O_3_ 262.1113, mp = 104–105 °C.

### 3.5. Reaction of 10 with 5-Bromo-2-aminopyridine Affording 1-(2,8-Dimethylimidazo[1,2-b]pyridazin-6-yl)ethan-1-one **14**

A mixture of 3-oxoester **10** (0.3 g, 1.15 mmol, 1.2 eq), 5-bromo-2-aminopyridine (0.17 g, 0.96 mmol, 1 eq), TsOH (0.4 g, 2.3 mmol, 2.4 eq), and toluene (5 mL) was stirred at reflux in a N_2_ atmosphere for 8 h (TLC—control on starting material, eluent—DCM:MeOH = 95:5 (by volume), R_f_ 0.45), cooled down to 20–22 °C, and filtered. The filter cake was washed with toluene (2 × 5 mL), suspended in a saturated aqueous solution of NaHCO_3_ (25 mL), and extracted with DCM (3 × 25 mL). The combined organic layers were washed with brine (2 × 30 mL), dried over anhydrous Na_2_SO_4_, and filtered. The filtrate was evaporated to dryness under reduced pressure to yield 1-(2,8-dimethylimidazo[1,2-*b*]pyridazin-6-yl)ethan-1-one 14 as a beige crystalline powder 90 mg (70%). ^1^H NMR (400 MHz, DMSO-*d*_6_, *δ*): 8.16 (^1^H, *s*, 7-CH), 7.48 (^1^H, *d*, *J* = 1.3 Hz, 3-CH), 2.64 (^3^H, *s*, CH_3_CO-), 2.58 (^3^H, *d*, *J* = 1.1 Hz, 2-CH_3_), 2.43 (^3^H, *s*, 8-CH_3_). ^13^C NMR (101 MHz, DMSO-*d*_6_, *δ*): 196.7 (6-COOCH_3_), 147.6 (C-6), 145.2 (C-9), 139.9 (C-2), 136.2 (C-8), 115.3 (C-3), 113.4 (C-7), 25.7 (6-COOCH_3_), 16.6 (2-CH_3_), 15.1 (8-CH_3_). HPLC-UV-MS (ESI+): found *m*/*z* 190.2 [M + H]^+^; calculated for C_10_H_12_N_3_O 190.1, mp = 140–141 °C.

### 3.6. Synthesis of Ethyl 3-Amino-3-(2,8-dimethylimidazo[1,2-b]pyridazin-6-yl)acrylate **15**

A stirred mixture of ethyl 3-(2,8-dimethylimidazo[1,2-*b*]pyridazine-6-yl)-3-oxopropionate **10** (42.3 g, 162 mmol, 1 eq) and toluene (150 mL) was heated to 80 °C to complete the solution, treated with NH_4_OAc (62.4 g, 810 mmol, 5 eq) and glacial AcOH (9 mL, 162 mmol, 1 eq), and heated at vigorous reflux for 4 h with a Dean–Stark trap and cooled down to 80 °C. More NH_4_OAc (62.4 g, 0.810 mol, 5 eq) was added and the whole was stirred at vigorous reflux for 2 h with a Dean–Stark trap (until total conversion of the starting material: TLC—control on product, eluent—DCM:MeOH = 95:5 (by volume), R_f_ 0.35). After the reaction was complete, the mixture was cooled to 20–20 °C, quenched with EtOAc (200 mL), and washed sequentially with saturated NaHCO_3_ aqueous solution (2 × 150 mL), deionized water (2 × 150 mL), and brine (2 × 150 mL). The organic layer was dried over anhydrous Na_2_SO_4_, filtered, and evaporated to dryness under reduced pressure. The residue was dissolved in *t*-BuOMe (100 mL) and the target ethyl 3-amino-3-(2,8-dimethylimidazo[1,2-*b*]pyridazine-6-yl)acrylate 15 was precipitated with *n*-hexane as a light beige powder, which was filtered off and air-dried to constant weight. Yield—31.6 g (81%). ^1^H NMR (400 MHz, DMSO-*d_6_*, *δ*): 8.02 (^1^H, *d*, *J* = 1.0 Hz, 7-CH), 7.84 (^1^H, *br.s.*, NH_2_), 7.51 (^1^H, *d*, *J* = 1.2 Hz, 3-CH), 7.26 (^1^H, *br.s.*, NH_2_), 5.30 (^1^H, s, =CH-), 4.10 (^2^H, *q*, *J* = 7.1 Hz, CH_2_CH_3_), 2.55 (^3^H, *d*, *J* = 1.2 Hz, 2-CH_3_), 2.40 (^3^H, *d*, *J* = 0.9 Hz, 8-CH_3_), 1.22 (^3^H, *t*, *J* = 7.1 Hz, CH_2_CH_3_). ^13^C NMR (101 MHz, DMSO-*d*_6_, *δ*): 169.3 (-CO_2_CH_2_CH_3_), 154.1 (6-C(NH_2_)=CHCO_2_Et), 146.0 (C-6), 143.6 (C-9), 138.9 (C-2), 135.7 (C-8), 114.5 (C-3), 113.8 (C-7), 83.5 (6-C(NH_2_)=CHCO_2_Et), 58.4 (-CO_2_CH_2_CH_3_), 16.1 (2-CH_3_), 14.5 (8-CH_3_), 14.4 (-CO_2_CH_2_CH_3_). HRMS (ESI+): found *m*/*z* 261.1373 [M + H]^+^; calculated for C_13_H_17_N_4_O_2_ 261.1273, mp = 93–94 °C.

### 3.7. Synthesis of 2-(2,8-Dimethylimidazo[1,2-b]pyridazin-6-yl)-7-fluoro-4H-pyrido[1,2-a]pyrimidin-4-one **16**

A mixture of ethyl 3-amino-3-(2,8-dimethylimidazo[1,2-*b*]pyridazine-6-yl)acrylate **15** (31.6 g, 122 mmol, 1 eq), 5-fluoro-2-iodopyridine (27.2 g, 122 mmol, 1 eq), K_2_CO_3_ (33.7 g, 244 mmol, 2 eq), CuI (4.7 g, 24.4 mmol, 0.2 eq), anhydrous 1,10-phenanthroline (6.6 g, 36.6 mol, 0.3 eq), and anhydrous DMF (100 mL) was vigorously stirred at 130 °C for 6 h (HPLC-UV-MS-control). After reaction completeness, the mixture was cooled down to 20–22 °C treated with deionized water (300 mL) and extracted with CHCl_3_ (3 × 150 mL). The combined organic layers were washed sequentially with deionized water (2 × 50 mL), brine (2 × 100 mL), dried over anhydrous Na_2_SO_4_, filtered, and stripped down from the solvent under diminished pressure. The residue was redissolved in a minimal amount of CHCl_3_ (90 mL) and the target compound, 2-(2,8-dimethylimidazo[1,2-*b*]pyridazin-6-yl)-7-fluoro-4*H*-pyrido[1,2-*a*]pyrimidin-4-one 16, was precipitated by addition of excess of hexane as a yellow-orange powder, which was collected by filtration and air-dried. Yield—26.3 g (81%). ^1^H NMR (400 MHz, CDCl_3_, *δ*): 8.96 (^1^H, *dd*, *J* = 4.5, 2.8 Hz, 6-CH), 7.85 (^1^H, *d*, *J* = 1.1 Hz, 7′-CH), 7.76–7.72 (^1^H, *m*, 9-CH), 7.73 (^1^H, *d*, *J* = 1.1 Hz, 3′-CH), 7.68–7.63 (^1^H, *m*, 8-CH), 7.38 (^1^H, *s*, 3-CH), 2.67 (^3^H, *d*, *J* = 1.1 Гц, 2′-CH_3_), 2.48 (^3^H, *d*, *J* = 0.9 Гц, 8′-CH_3_). ^13^C NMR (101 MHz, CDCl_3_, *δ*): 157.8 (C-4, *d*, *J_(C-F)_* = 2.1 Hz), 157.5 (C-6′), 154.3 (C-7, *d*, *J_(C-F)_* = 247.0 Hz), 149.1 (C-10), 147.9 (C-2), 144.5 (C-9′), 140.0 (C-2′), 135.8 (C-8′), 129.1 (C-8, *d*, *J_(C-F)_* = 25.4 Hz), 128.6 (C-9, *d*, *J_(C-F)_* = 7.2 Hz), 114.8 (C-7′), 114.7 (C-3′), 113.8 (C-6, *d*, *J_(C-F)_* = 41.0 Hz), 100.1 (C-3), 16.9 (2′-CH_3_), 14.9 (8′-CH_3_). HRMS (ESI+): found *m*/*z* 310.1099 [M + H]^+^; calculated for C_16_H_13_FN_5_O 310.1026, mp = 268–269 °C (d).

### 3.8. Synthesis of Tert-Butyl 7-(2-(2,8-Dimethylimidazo[1,2-b]pyridazin-6-yl)-4-oxo-4H-pyrido[1,2-a]pyrimidin-7-yl)-4,7-diazaspiro[2.5]octane-4-carboxylate **17**

A solution of 2-(2,8-dimethylimidazo[1,2-*b*]pyridazine-6-yl)-7-fluoro-4*H*-pyrido[1,2-*a*]pyrimidine-4-one 16 (26.3 g, 85 mmol, 1 eq) in dimethylacetamide (DMAc, 80 mL) was treated with *tert*-butyl 4,7-diazaspiro[2.5]octane-4-carboxylate obtained as described earlier [19] (18 g, 85 mmol, 1 eq) and DBU (14 mL, 94 mmol, 1.1 eq) and was subsequently stirred under protection of atmospheric moisture and CO_2_ at 120 °C until the total consumption of the starting material (HPLC-UV-MS-control). The reaction mixture was cooled down to 20–22 °C and stirred into 500 mL of deionized water and the obtained precipitate was filtered off and dried to constant weight at reduced pressure to produce the target *tert*-butyl 7-(2-(2,8-dimethylimidazo[1,2-*b*]pyridazine-6-yl)-4-oxo-4*H*-pyrido[1,2-*a*]pyrimidine-7-yl)-4,7-diazaspiro[2.5]octane-4-carboxylate 17 as a light yellow powder. Yield—15 g (42%). ^1^H NMR (400 MHz, CDCl_3_, *δ*): 8.36 (^1^H, *d*, *J* = 2.5 Hz, 6′-CH), 7.84 (^1^H, *d*, *J* = 1.2 Hz, 7′′-CH), 7.72 (^1^H, *d*, *J* = 1.0 Hz, 3′′-CH), 7.67–7.63 (^1^H, *m*, 9′-CH), 7.63–7.59 (^1^H, *m*, 8′-CH), 7.30 (^1^H, *s*, 3′-CH), 3.70 (^2^H, *dd*, *J* = 6.1, 4.1 Hz, 6-CH_2_), 3.21 (^2^H, *t*, *J* = 5.1 Hz, 5-CH_2_), 3.00 (^2^H, *s*, 8-CH_2_), 2.66 (^3^H, *d*, *J* = 1.1 Hz, 8′′-CH_3_), 2.47 (^3^H, *d*, *J* = 0.8 Hz, 2′′-CH_3_), 1.42 (^9^H, *s*, -CO_2_C(CH_3_)_3_), 1.06–1.03 (^2^H, *m*, 1-CH_2_, 2-CH_2_), 0.84–0.80 (^2^H, *m*, 1-CH_2_, 2-CH_2_). ^13^C NMR (101 MHz, CDCl_3_, *δ*): 158.1 (C-4′), 156.3 (C-6′′), 155.4 (-CO_2_C(CH_3_)_3_), 148.5 (C-2′), 147.2 (C-10′), 144.1 (C-2′′), 141.9 (C-7′), 140.0 (C-9′′), 135.6 (C-8′′), 130.9 (C-8′), 126.8 (C-9′), 114.9 (C-7′′), 114.7 (C-3′′), 110.1 (C-6′), 99.3 (C-3′), 80.5 (-CO_2_C(CH_3_)_3_), 55.2 (C-8), 48.0 (C-5), 45.3 (C-6), 37.4 (C-3), 28.4 (-CO_2_C(CH_3_)_3_), 16.9 (8′′-CH_3_), 14.9 (2′′-CH_3_), 14.2 (C-1, C-2). HRMS (ESI+): found *m*/*z* 502.2576 [M + H]^+^; calculated for C_27_H_31_N_7_O_3_ 502.2488, mp = 221–222 °C.

### 3.9. Synthesis of 2-(2,8-Dimethylimidazo[1,2-b]pyridazin-6-yl)-7-(4,7-diazaspiro[2.5]octan-7-yl)-4H-pyrido[1,2-a]pyrimidin-4-one Trihydrate **18**

AcCl (85 mL, 1.218 mol, 7 eq) was dissolved in *n*-BuOH (250 mL), and the resulting solution was heated to 65 °C and mixed with the pre-heated sample to the same temperature solution of *tert*-butyl 7-(2-(2,8-dimethylimidazo[1,2-*b*]pyridazin-6-yl)-4-oxo-4*H*-pyrido[1,2-*a*]pyrimidin-7-yl)-4,7-diazaspiro[2.5]octane-4-carboxylate 17 (15 g, 30 mmol, 1 eq) in *n*-BuOH (150 mL), giving a suspension. After stirring the reaction mixture until the total consumption of the starting material (HPLC-UV-MS-control), the precipitate was filtered off, washed with cold *n*-BuOH (3 × 100 mL), and dried in high vacuum to obtain the corresponding dihydrochloride 2-(2,8-dimethylimidazo[1,2-*b*]pyridazine-yl)-7-(4,7-diazospiro[2.5]octane-7-yl)-4*H*-pyrido[1,2-*a*]pyrimidine-4-one as a yellow powder. Subsequently, obtained dihydrochloride was dissolved in deionized water (150 mL) and treated with 95% EtOH (150 mL). The solution was adjusted to pH = 13 by dropwise addition of 32% NaOH aqueous solution (3 mL, approx. 32.5 mmol, 1.3 eq), leading to the formation of the yellow precipitate. The resulting suspension was vigorously stirred at 50 °C for 6 h, gradually cooled to 20–22 °C, and filtered. A filter cake was thoroughly washed with 30% aqueous EtOH and the precipitate was dried in vacuo at 50 °C to a constant weight, yielding 10.2 g (85%) of 2-(2,8-dimethylimidazo[1,2-*b*]pyridazine-6-yl)-7-(4,7-diazospiro[2.5]octane-7-yl)-4*H*-pyrido[1,2-*a*]pyrimidine-4-one trihydrate 18. ^1^H NMR (400 MHz, CDCl_3_, *δ*): 8.36 (^1^H, *d*, *J* = 2.4 Hz, 6-CH), 7.84 (^1^H, *q*, *J* = 1.3 Hz, 7′-CH), 7.72 (^1^H, *s*, 3′-CH), 7.66–7.63 (^1^H, *m*, 9-CH), 7.63–7.60 (^1^H, *m*, 8-CH), 7.29 (^1^H, *s*, 3-CH), 3.19–3.16 (^2^H, *m*, 6′′-CH_2_), 3.12–3.09 (^2^H, *m*, 5′′-CH_2_), 3.00 (^2^H, *s*, 8′′-CH_2_), 2.66 (^3^H, *s*, 8′-CH_3_), 2.47 (^3^H, *s*, 2′-CH_3_), 0.69–0.66 (^2^H, *m*, 1′′-CH_2_, 2′’-CH_2_), 0.59–0.56 (^2^H, *m*, 1′′-CH_2_, 2′′-CH_2_). ^13^C NMR (101 MHz, CDCl_3_, *δ*): 158.1 (C-4), 156.2 (C-6′), 148.5 (C-2), 147.2 (C-10), 144.1 (C-2′), 142.2 (C-7), 140.0 (C-9′), 135.6 (C-8′), 131.2 (C-8), 126.7 (C-9), 114.9 (C-7′), 114.7 (C-3′), 110.0 (C-6), 99.2 (C-3), 56.7 (C-8′′), 49.9 (C-5′′), 44.5 (C-6′′), 36.5 (C-3′′), 16.9 (8′-CH_3_), 14.9 (2′-CH_3_), 13.1 (C-1′′, C-2′′). HRMS (ESI+): found *m*/*z* 402.2051 [M + H]^+^; calculated for C_22_H_24_N_7_O 402.1964, mp = 269–270°C (d).

## 4. Conclusions

In this work, we have developed an efficient, straightforward, and environmentally conscious synthetic route to risdiplam, a clinically significant small-molecule therapy for spinal muscular atrophy. Previously developed and optimized procedures for the synthesis of the challenging intermediates **7** and **9** enabled the design of the novel and convenient 5-step approach (starting from building block **11**) to the synthesis of the target molecule, based upon the cuprous iodide complex-catalyzed heterocyclization reaction. For the first time, the core heterocycle **16** is constructed without relying on Pd complex-catalyzed reactions, enabling the preparation of risdiplam with high purity (99.86%, by HPLC-UV with a maximum content of a single, unidentified impurity not exceeding 0.046%) and a 20% overall yield (from **11**) without utilizing chromatographic purification. By significantly reducing the need for chlorinated solvents and an amount of inorganic waste, enabling tight purity control at key check-points, and improving atom economy, this route addresses critical drawbacks of earlier strategies and offers a scalable alternative for the sustainable production of risdiplam.

## Figures and Tables

**Table 1:** Selection of the compound **12** synthesis conditions.

Substrate	Metal Source	Ligand	Base	Solvent	T °C	Yield
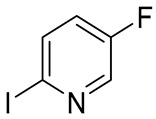	CuI (20 mol%)	No ligand	K_2_CO_3_ (2 eq)	DMF	130 °C	29%
		PPh_3_ (30 mol%)				58%
		MePhos (30 mol%)				64%
		1,10-Phenanthroline (30 mol%)				**81%**
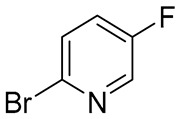	CuI (20 mol%)	MePhos (30 mol%)				47%
		1,10-Phenanthroline (30 mol%)				62%
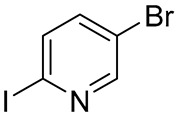	CuI (20 mol%)	MePhos (30 mol%)				a mixture of mono- and di-halogen-substituted products ^1^
		1,10-Phenanthroline (30 mol%)				

^1^ Impurities detection and characterization via HPLC-UV-MS analysis.

## Data Availability

The original contributions presented in this study are included in the article/Supplementary Material. Further inquiries can be directed to the corresponding author(s).

## Supplementary Materials

The following supporting information can be downloaded at: https://www.mdpi.com/article/10.3390/molecules30163375/s1. Copies of ^1^H- and ^13^C-NMR spectra, HRMS spectra for all compounds obtained, as well as results of analysis of the risdiplam obtained purity.
