# Supplementary material for: A Convenient, Pd-Free Approach to the Synthesis of Risdiplam
Source: Molecules. 2025 Aug 14;30(16):3375. doi: 10.3390/molecules30163375 (PMC12388427; doi:10.3390/molecules30163375)
Supplement: Supplementary file 1 [file molecules-30-03375-s001.zip › molecules-3798372-supplementary.pdf]

ELECTRONIC SUPPLEMENTARY MATERIAL

A Convenient, Pd-Free Approach to the Synthesis of Risdiplam

Georgiy Korenev *, Alexey A. Gutenev, Fyodor V. Antipin, Vladimir V. Chernyshov, Julia A. Shulgina, Maria P. Korobkina, Maxim B. Nawrozkij and Roman A. Ivanov

Medicinal Biotechnology Department, Sirius University of Science and Technology, Olimpiyskiy Ave. 1, 354340 Sirius, Krasnodar Region, Russia; vladimir.chernyshov2012@yandex.ru (V.V.C.); navrotskij.mb@talantiuspeh.ru (M.B.N.); ivanov.ra@talantiuspeh.ru (R.A.I.)

* Correspondence: korenev.g@talantiuspeh.ru

Table of Contents

Copies of NMR spectra 3
Copies of HRMS spectra 15
Analysis of the risdiplam purity 20

Copies of NMR spectra

^{1}H NMR spectrum of ethyl 3-(2,8-dimethylimidazo[1,2-b]pyridazin-6-yl)-3-oxopropanoate **10**

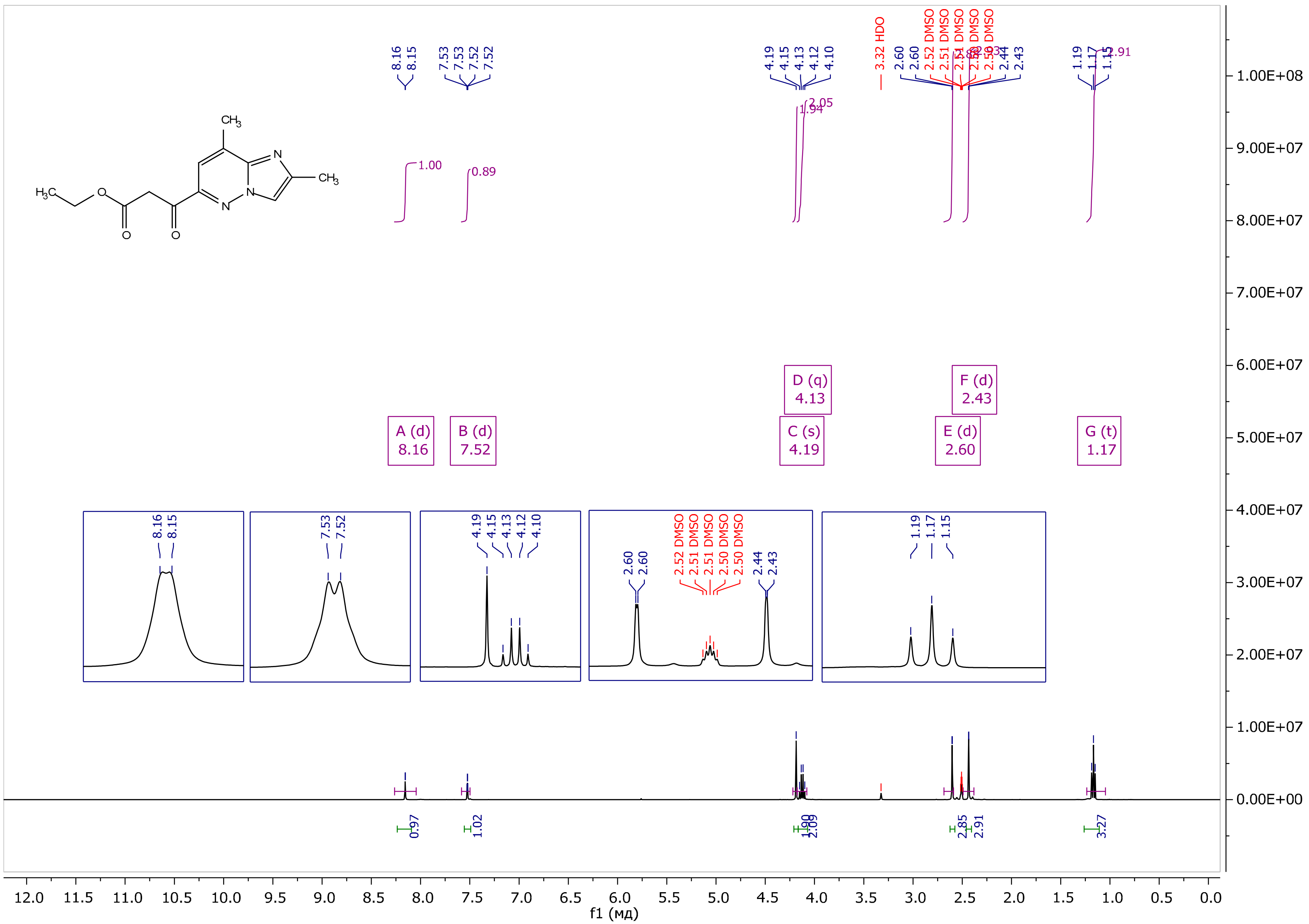

^{13}C NMR spectrum of ethyl 3-(2,8-dimethylimidazo[1,2-b]pyridazin-6-yl)-3-oxopropanoate **10**

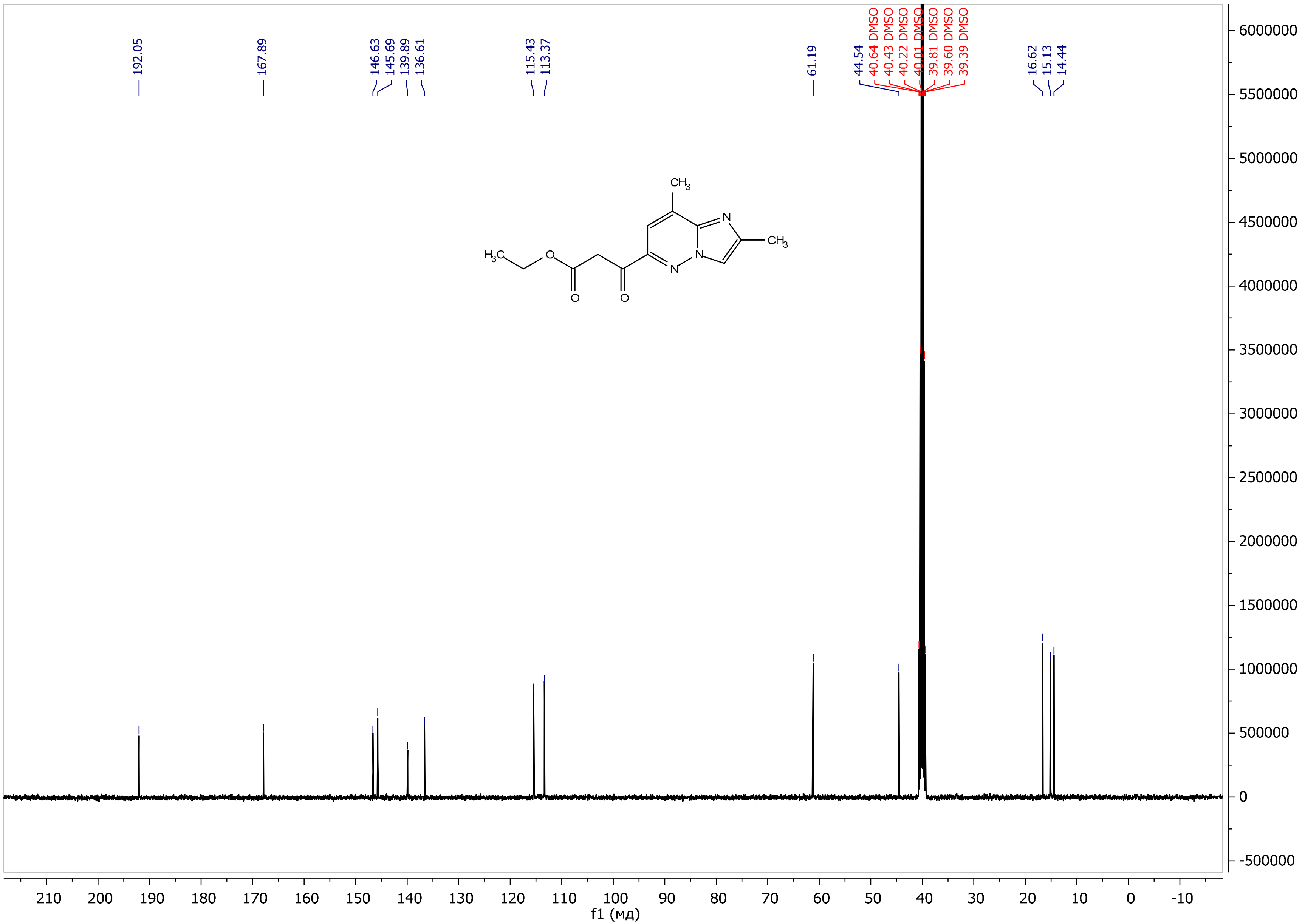

^{1}H NMR spectrum of 1-(2,8-dimethylimidazo[1,2-b]pyridazin-6-yl)ethan-1-one **14**

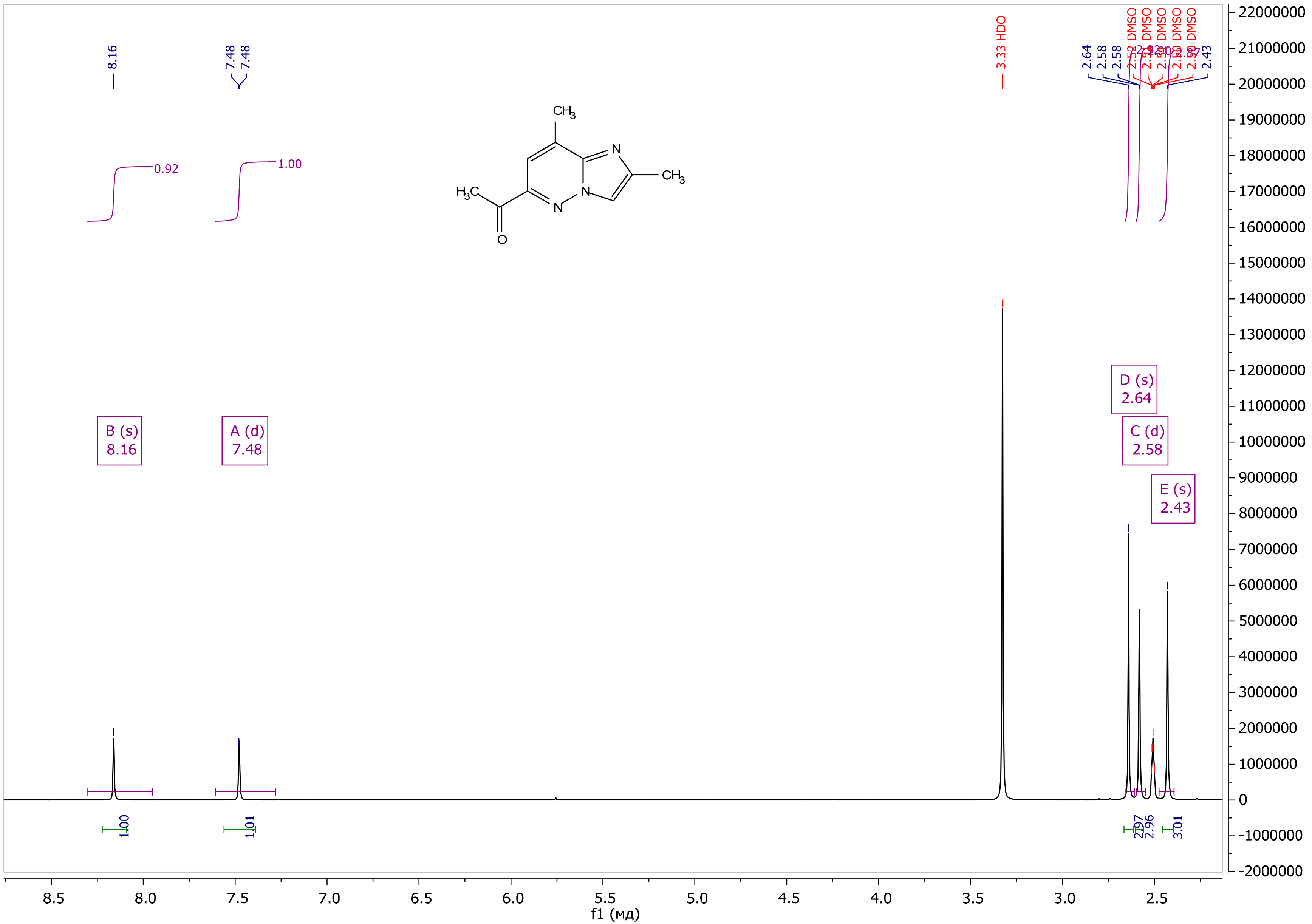

^{13}C NMR spectrum of 1-(2,8-dimethylimidazo[1,2-b]pyridazin-6-yl)ethan-1-one **14**

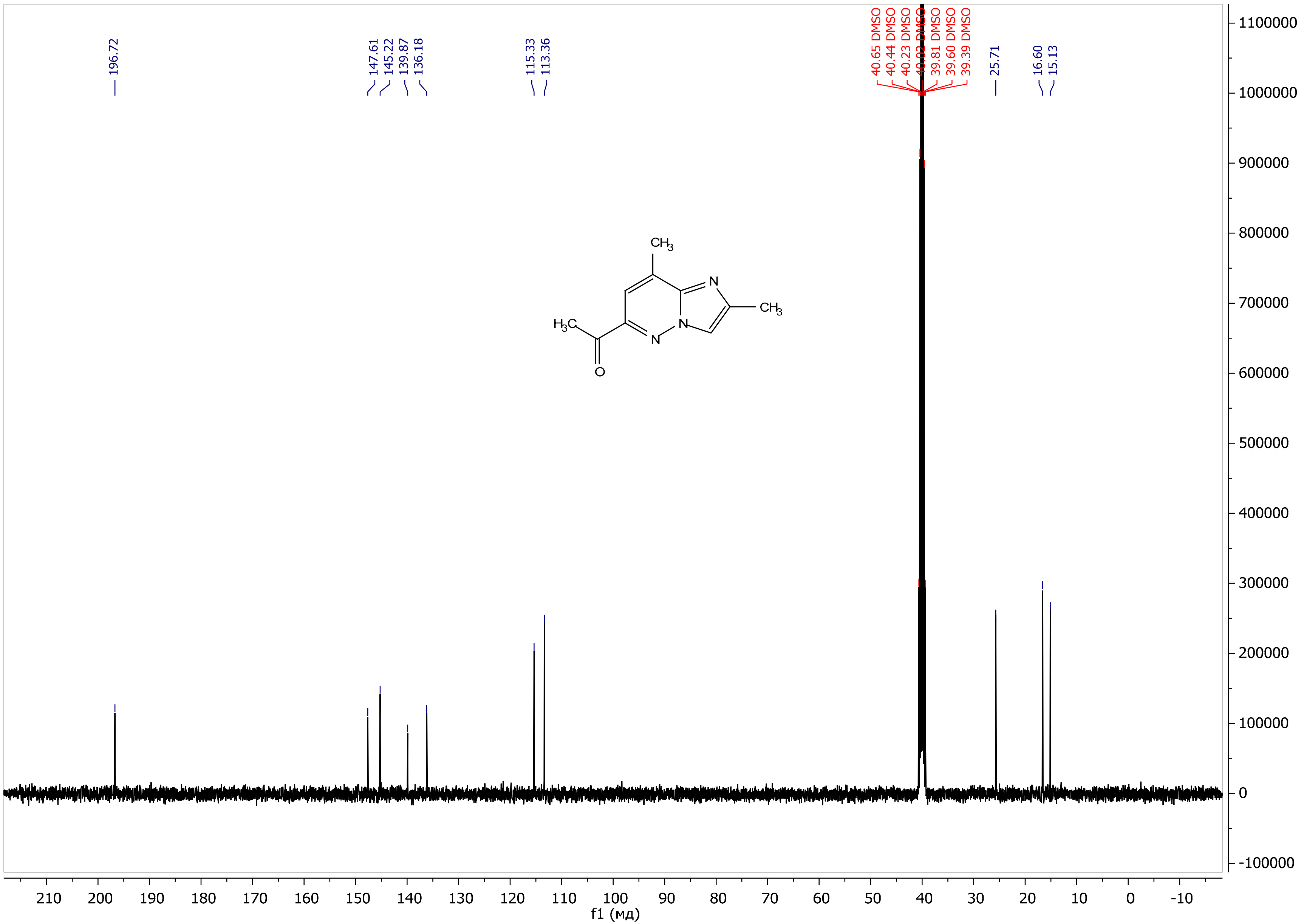

^{1}H NMR spectrum of ethyl 3-amino-3-(2,8-dimethylimidazo[1,2-b]pyridazin-6-yl)acrylate **15**

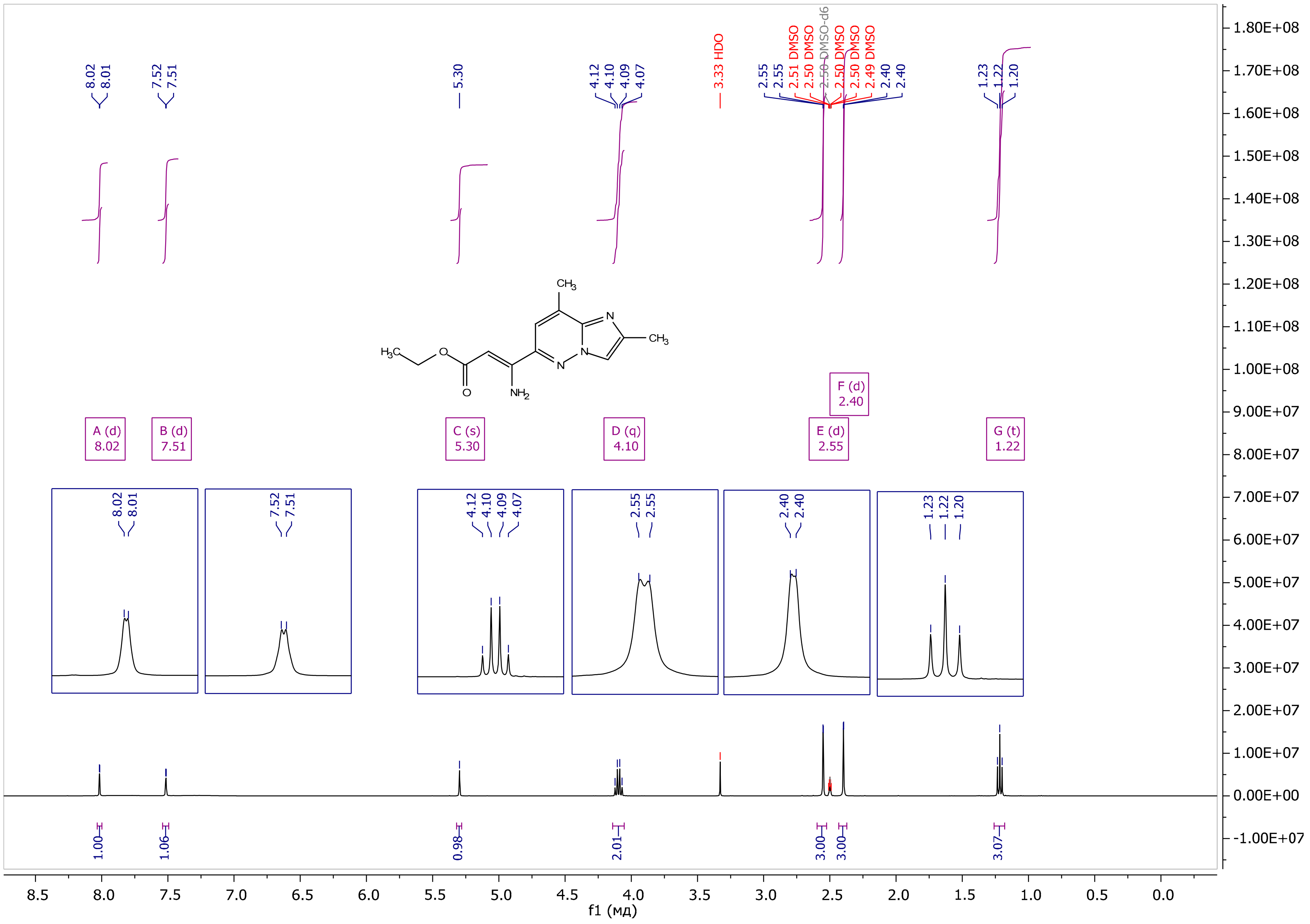

^{13}C NMR spectrum of ethyl 3-amino-3-(2,8-dimethylimidazo[1,2-b]pyridazin-6-yl)acrylate **15**

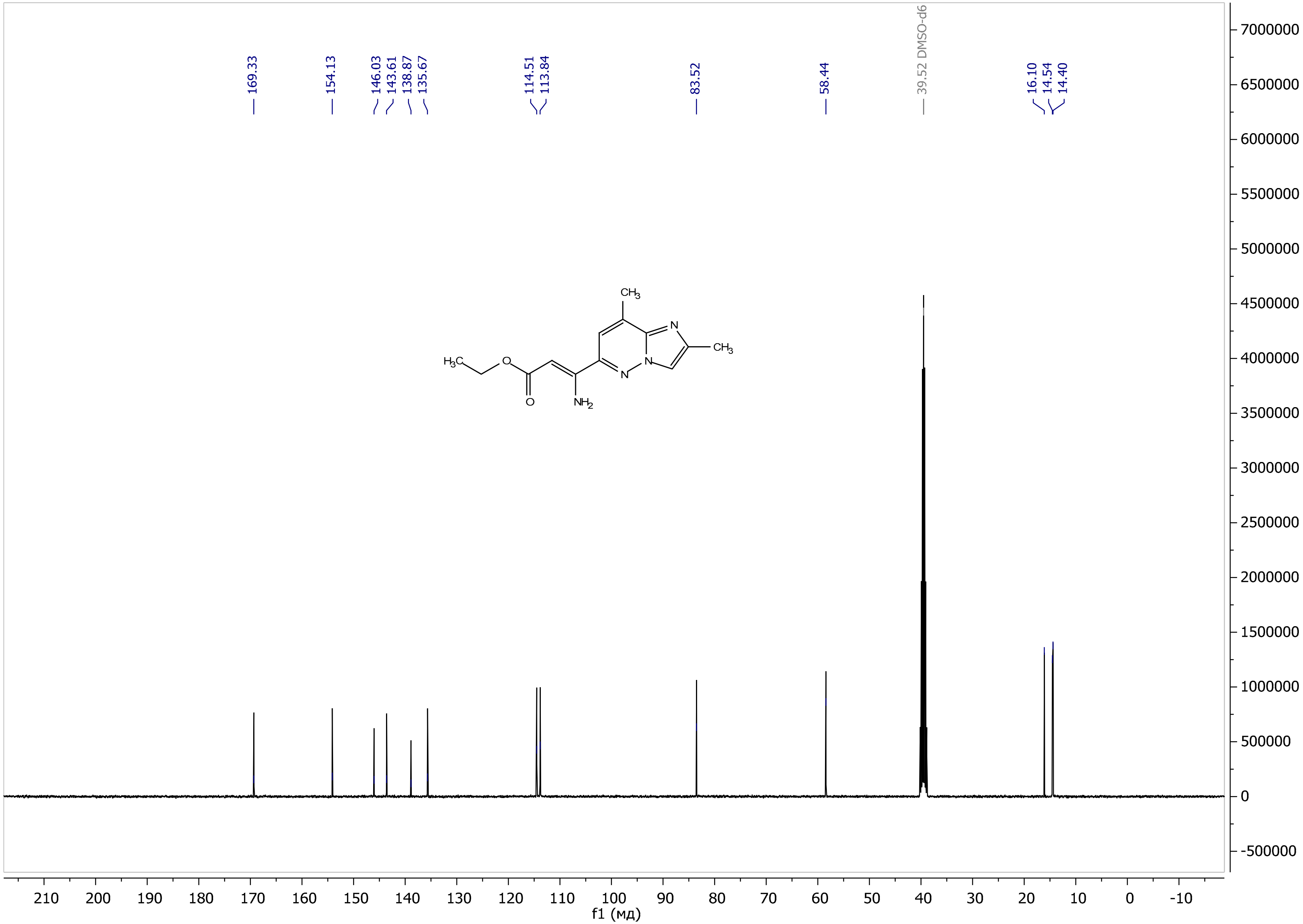

^{1}H NMR spectrum of 2-(2,8-dimethylimidazo[1,2-b]pyridazin-6-yl)-7-fluoro-4H-pyrido[1,2-a]pyrimidin-4-one **16**

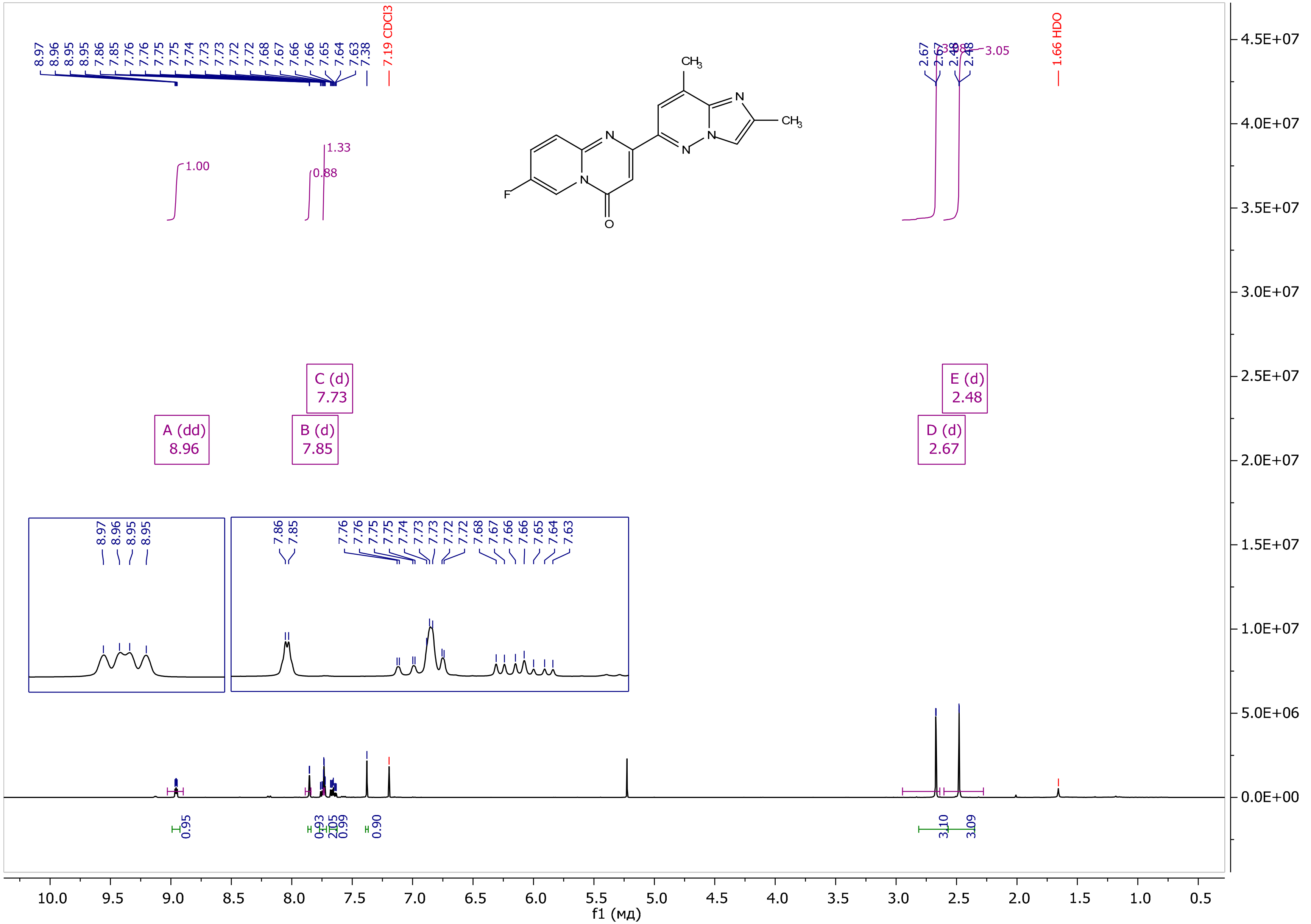

^{13}*C NMR spectrum of 2-(2,8-dimethylimidazo[1,2-b]pyridazin-6-yl)-7-fluoro-4H-pyrido[1,2-a]pyrimidin-4-one* **16**

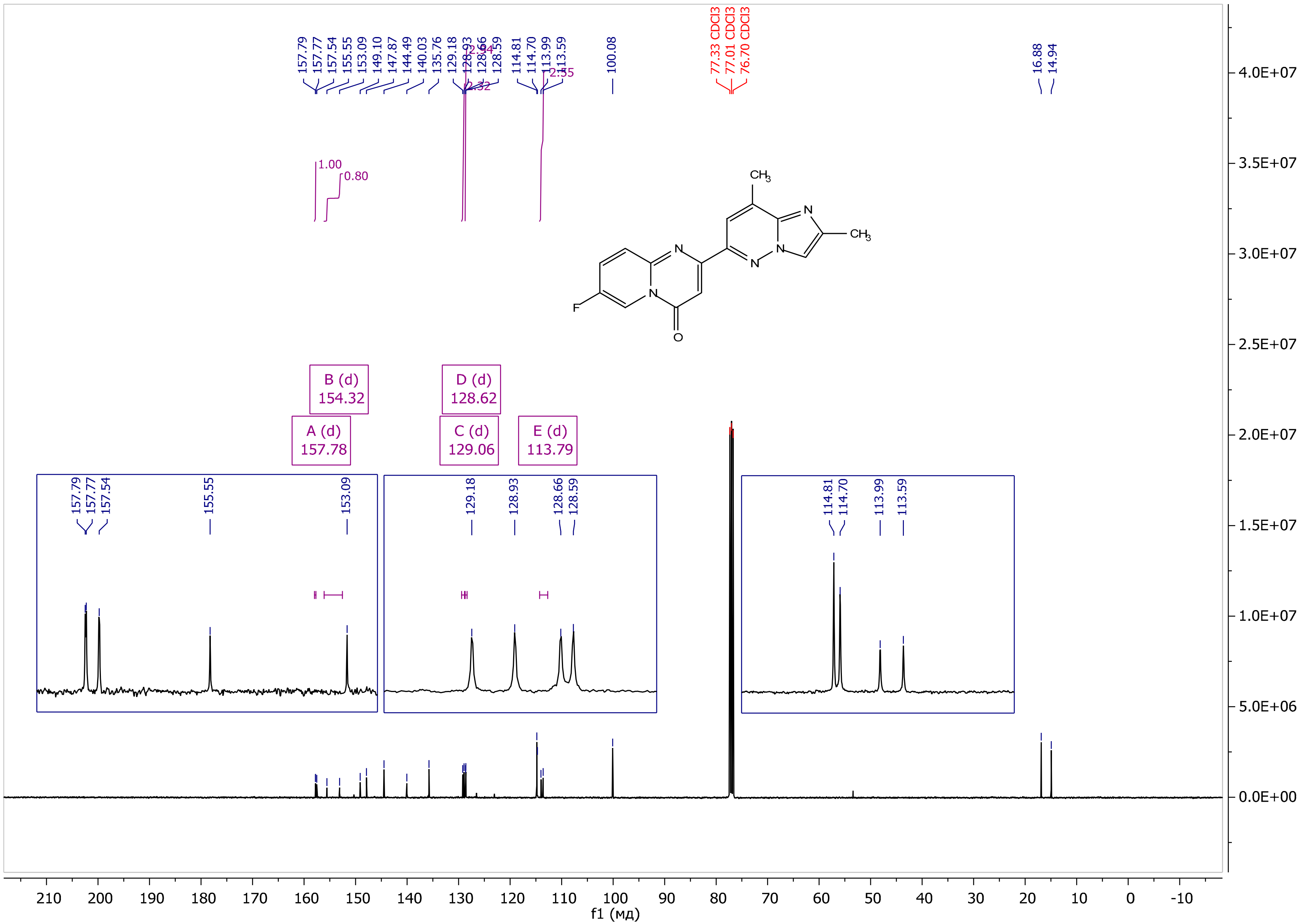

^{1}H NMR spectrum of tert-butyl 7-(2-(2,8-dimethylimidazo[1,2-b]pyridazin-6-yl)-4-oxo-4H-pyrido[1,2-a]pyrimidin-7-yl)-4,7-diazaspiro[2.5]octane-4-carboxylate **17**

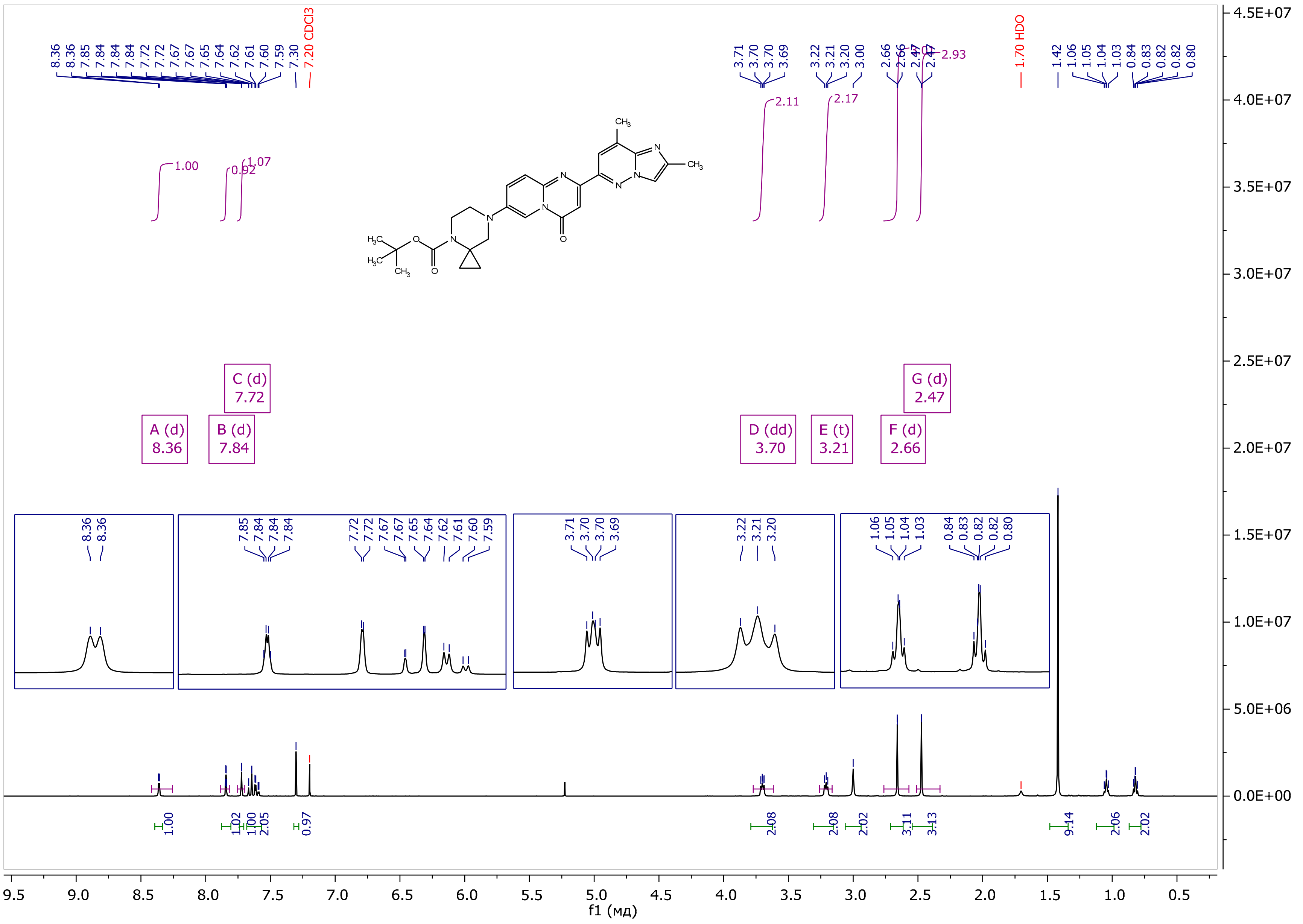

^{13}C NMR spectrum of *tert-butyl 7-(2-(2,8-dimethylimidazo[1,2-b]pyridazin-6-yl)-4-oxo-4H-pyrido[1,2-a]pyrimidin-7-yl)-4,7-diazaspiro[2.5]octane-4-carboxylate* **17**

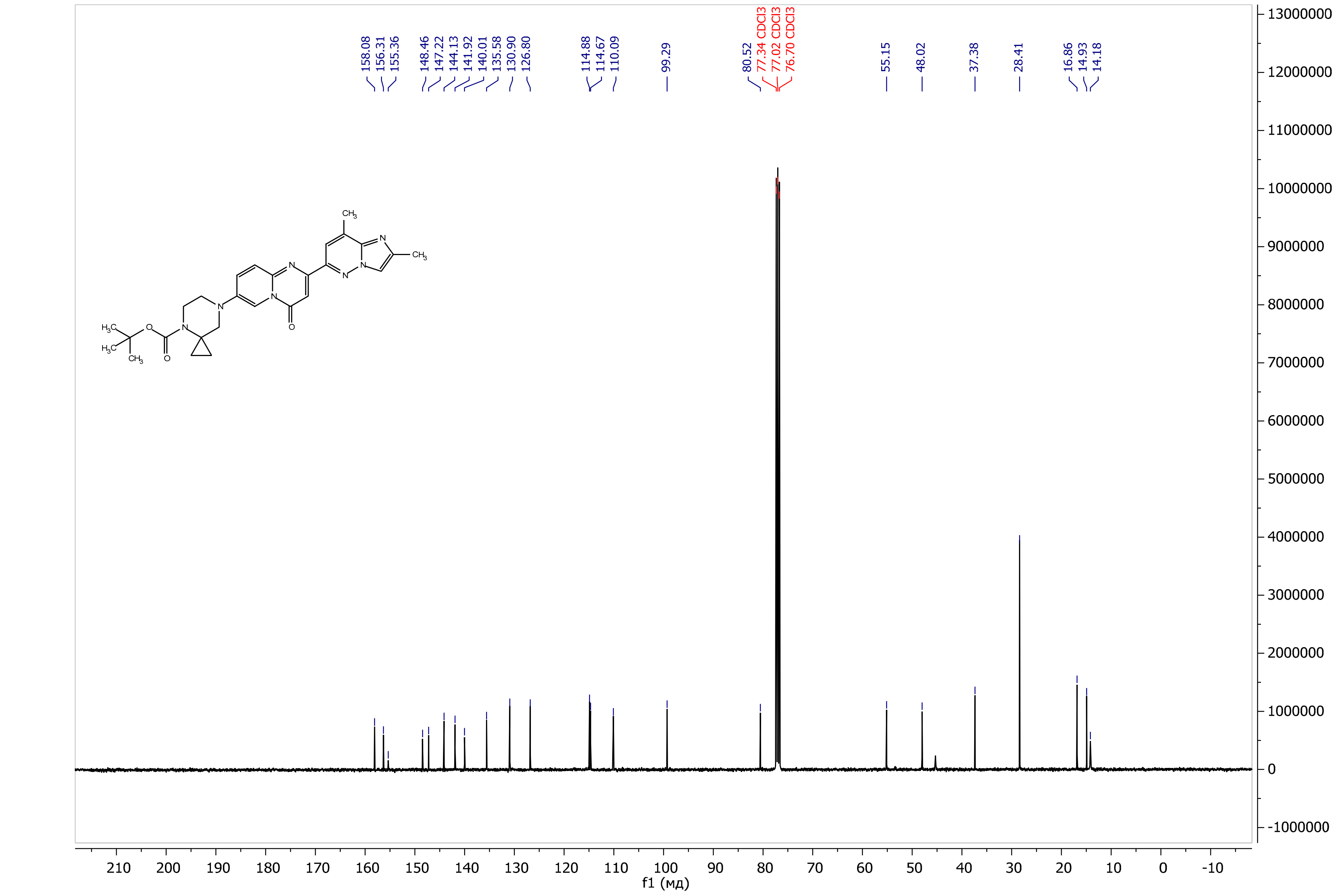

^{1}H NMR spectrum of 2-(2,8-dimethylimidazo[1,2-b]pyridazin-6-yl)-7-(4,7-diazaspiro[2.5]octan-7-yl)-4H-pyrido[1,2-a]pyrimidin-4-one (Risdiplam) **18**

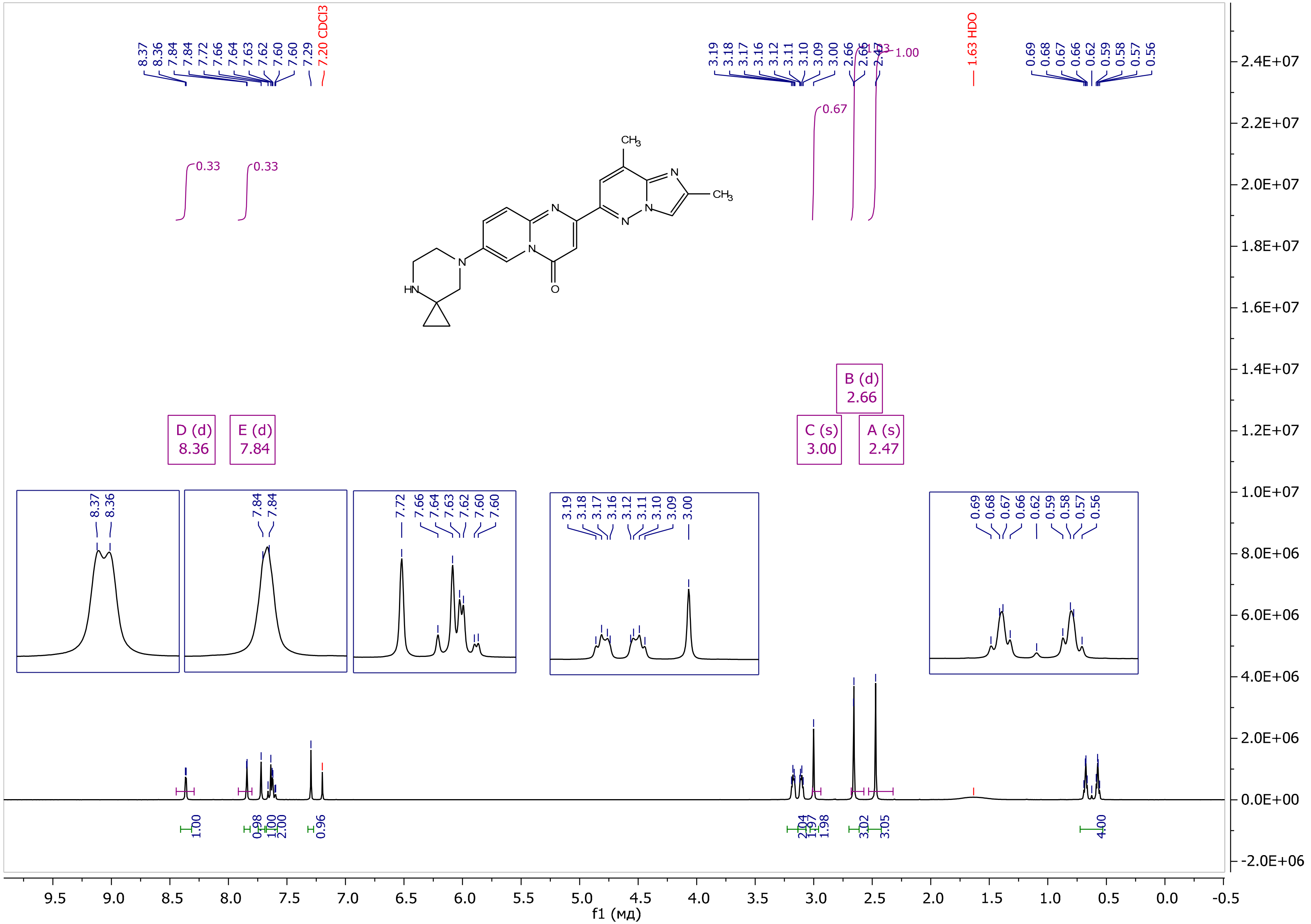

^{13}C NMR spectrum of 2-(2,8-dimethylimidazo[1,2-b]pyridazin-6-yl)-7-(4,7-diazaspiro[2.5]octan-7-yl)-4H-pyrido[1,2-a]pyrimidin-4-one (Risdiplam) **18**

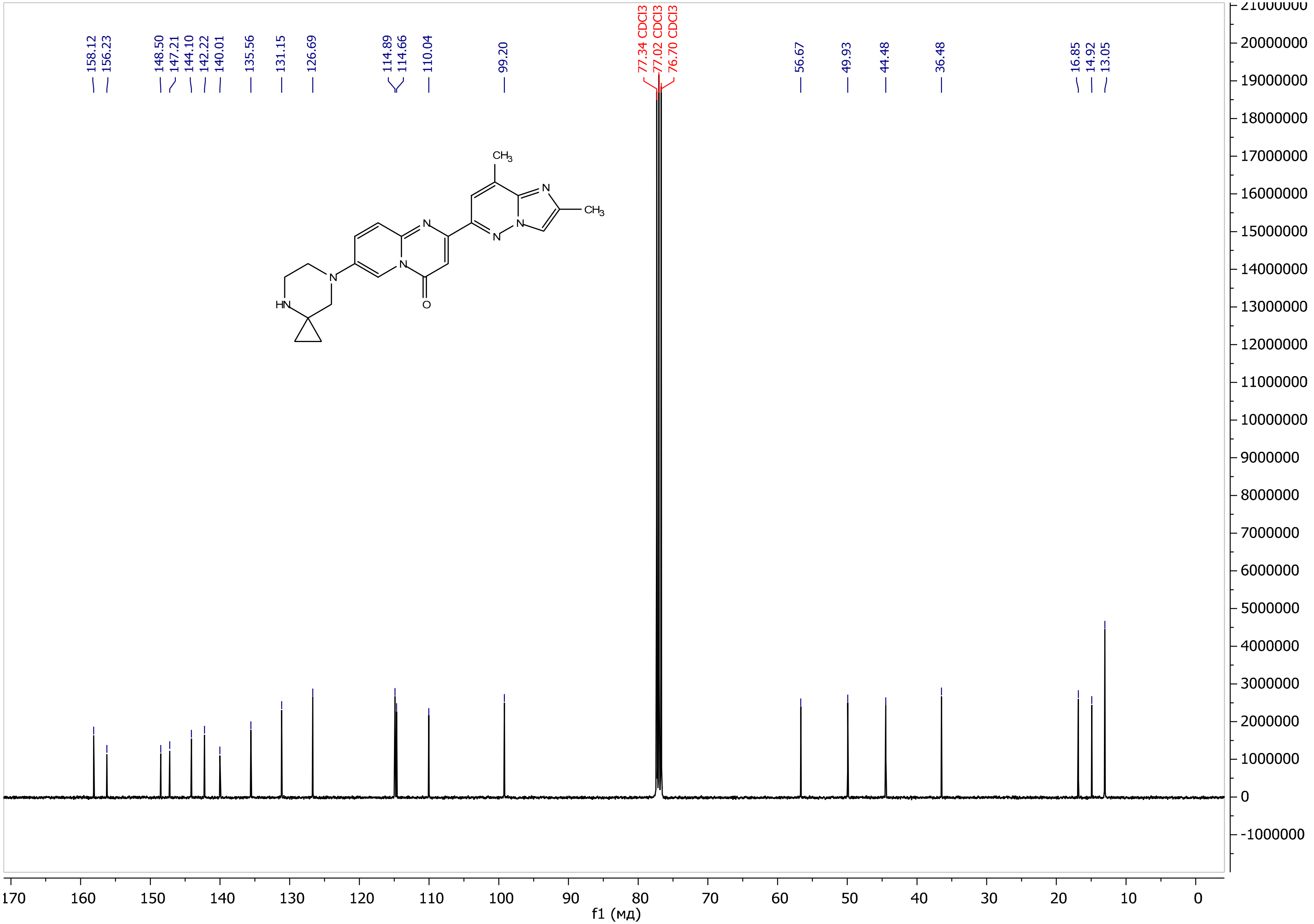

Copies of HRMS spectra

Compound	Structure	HRMS spectra (MS and MS2, correspondingly)
10		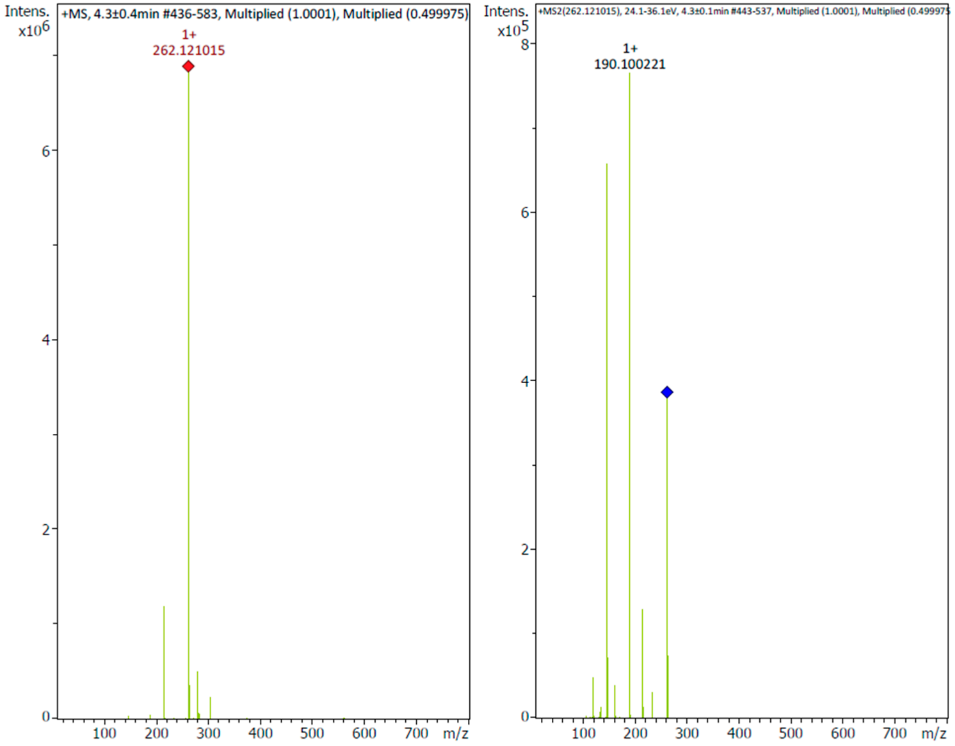

**15**			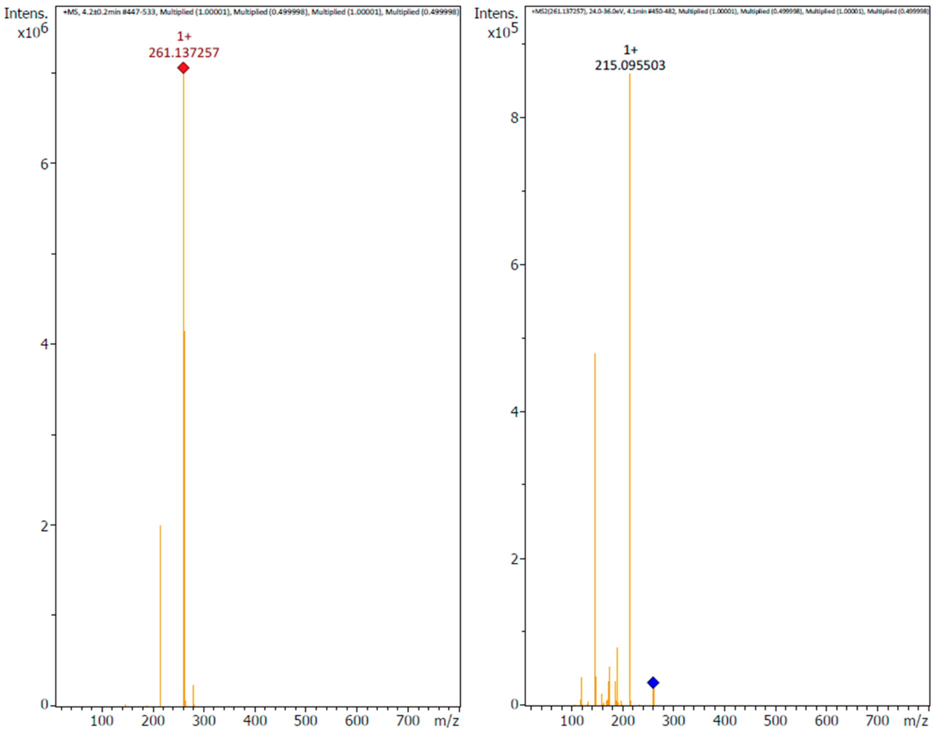 Intens.<br>x10^6<br>1+<br>261.137257<br>0<br>2<br>4<br>6<br>100 200 300 400 500 600 700 m/z<br>Intens.<br>x10^5<br>1+<br>215.095503<br>0<br>2<br>4<br>6<br>8<br>100 200 300 400 500 600 700 m/z

**16**		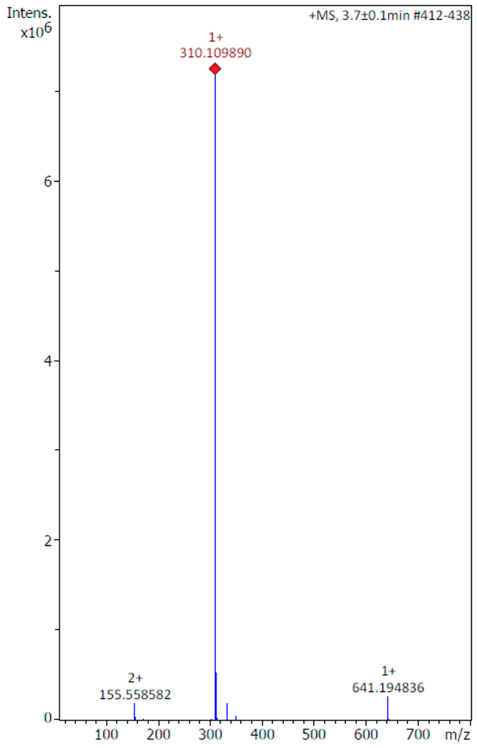 Intens. x10^6<br>+MS, 3.7±0.1min #412-438<br>1+ 310.109890<br>2+ 155.558582<br>1+ 641.194836<br>m/z 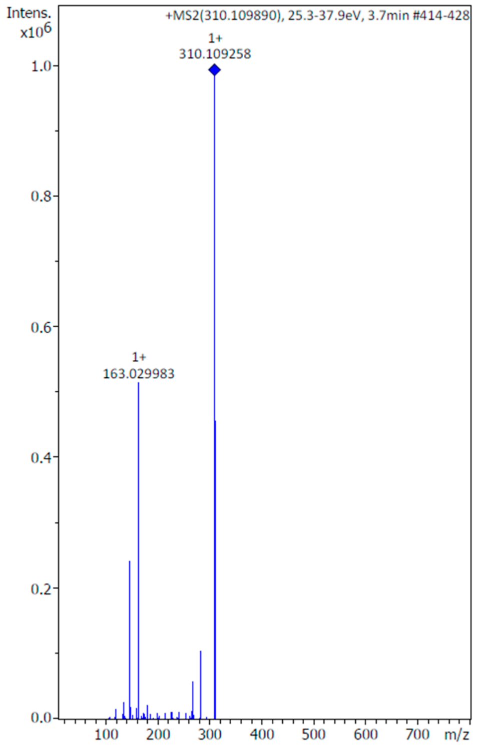 Intens. x10^6<br>+MS2(310.109890), 25.3-37.9eV, 3.7min #414-428<br>1+ 310.109258<br>1+ 163.029983<br>m/z

**17**		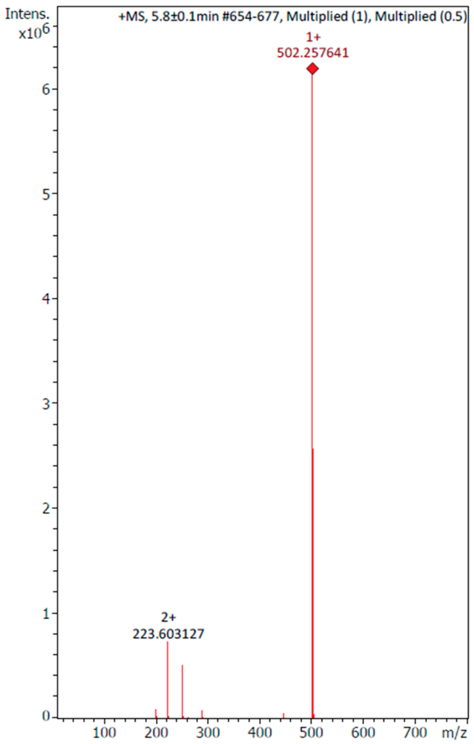Intens.<br>x10^6<br>+MS, 5.8±0.1min #654-677, Multiplied (1), Multiplied (0.5)<br>1+<br>502.257641<br>2+<br>223.603127<br>m/z 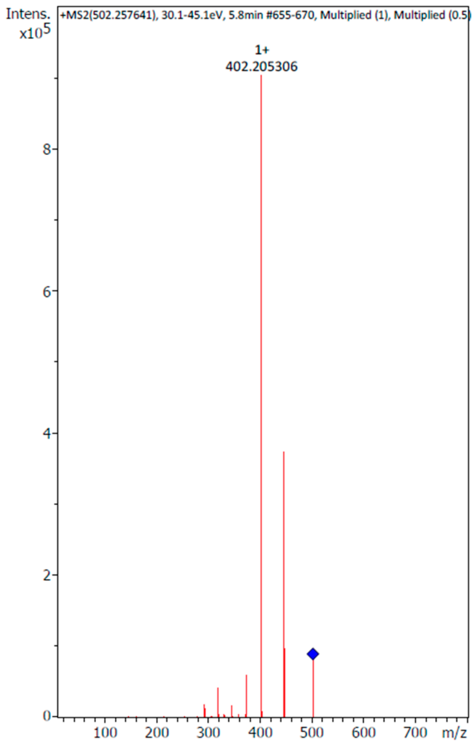Intens.<br>x10^5<br>+MS2(502.257641), 30.1-45.1eV, 5.8min #655-670, Multiplied (1), Multiplied (0.5)<br>1+<br>402.205306<br>m/z

**18**			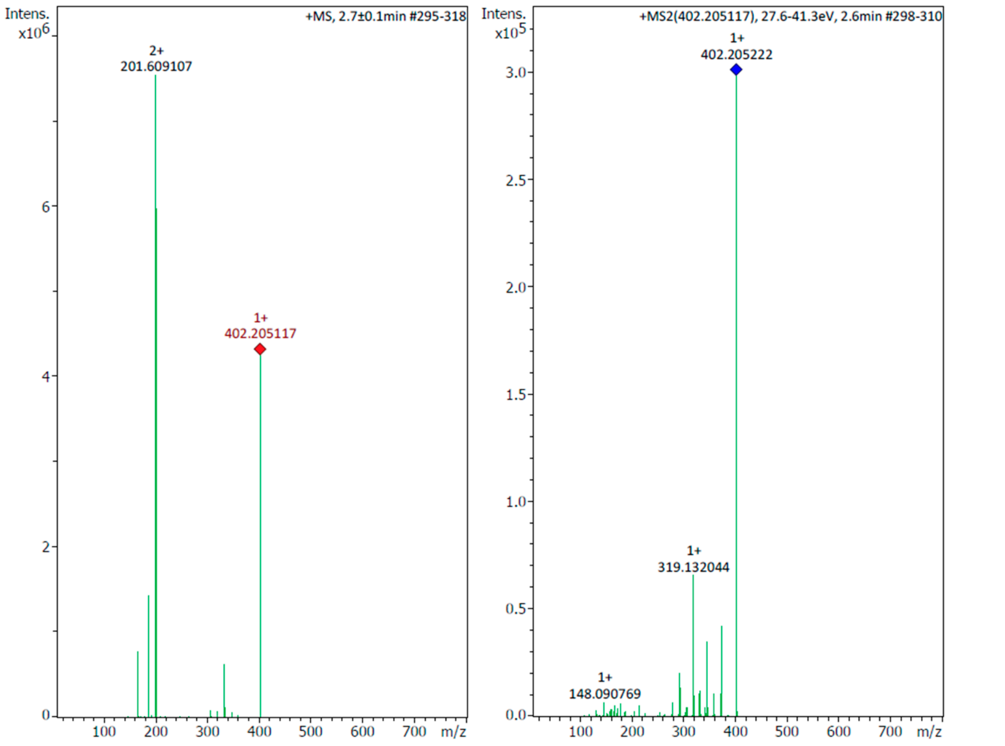

Intens.
x10^6
+MS, 2.7±0.1min #295-318
2+
201.609107
1+
402.205117
0
2
4
6
100
200
300
400
500
600
700
m/z
Intens.
x10^5
+MS2(402.205117), 27.6-41.3eV, 2.6min #298-310
1+
402.205222
1+
319.132044
1+
148.090769
0.0
0.5
1.0
1.5
2.0
2.5
3.0
100
200
300
400
500
600
700
m/z

Analysis of the risdiplam purity

SHIMADZU LabSolutions

Analysis Report

Name of the sample : Ris_3H2O_0603
Sample number : 07.03.2025
Name of the data file : Ris_3H2O_0603.lcd
Date of processing : 11.03.2025 9:45:41
Method file name : Poroshell 120 EC-C18 gradient_RID_A_D_256нм_258нм_0,5 мл_30гр_27пр_40мин.lcm
File series name : 07.03.2025.lcb
Vial number : 3-16
Injection volume : 3 uL
Date of receipt : 07.03.2025 18:07:32

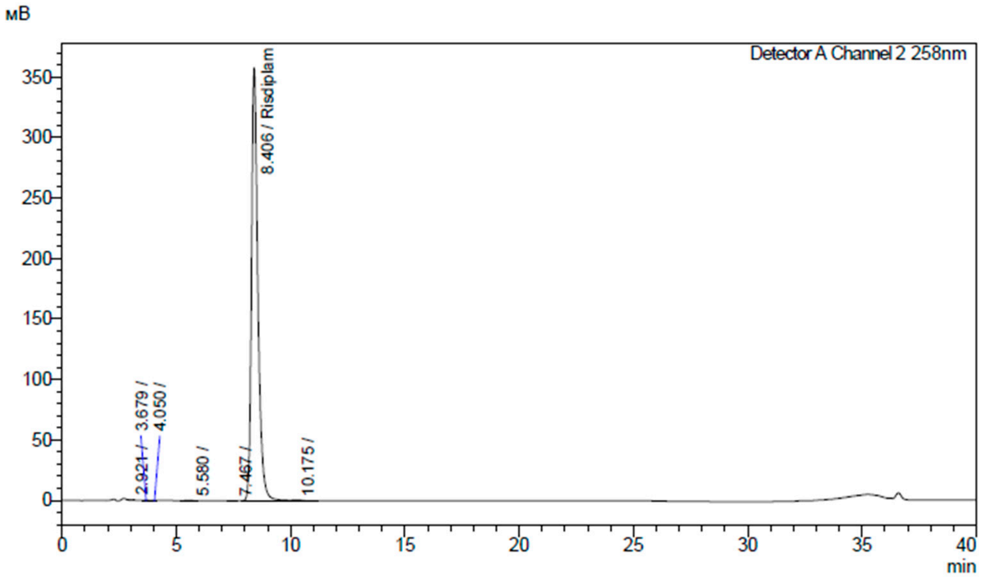

Detector A Channel 2 258nm

Name	Ret. Time	Area	Height	N(USP)	AS
	2.921	1539	171	2317	--
	3.679	3234	273	3071	--
	4.050	369	37	2516	--
	5.580	3154	176	10292	0.982
	7.467	512	39		0.908
Risdiplam	8.406	6949801	357840	4716	1.367
	10.175	1183	60	8118	2.315
		6959793	358596		
